# Isolation and identification of *Aeromonas salmonicida* of bovine origin and its genome-wide and pathogenicity analysis

**DOI:** 10.3389/fmicb.2025.1712790

**Published:** 2026-01-12

**Authors:** Qian Wang, Xiaoping Dong, Miaomiao Cui, Xuechao Liang, Yiya Sun, Shulin Chen, Shuo Feng, Xiwen Zheng, Yingzi Liu

**Affiliations:** 1 College of Animal Husbandry and Veterinary, Jinzhou Medical University, Jinzhou, China; 2 Department of Clinical Laboratory, Affiliated First Hospital of Jinzhou Medical University, Jinzhou, China

**Keywords:** *Aeromonas salmonicida*, isolation and identification, genome-wide sequencing, pathogenicity, cross-species, drug resistance

## Abstract

*Aeromonas salmonicida* subsp. *pectinolytica* is a type of bacterium capable of growing at 37 °C, and it was traditionally considered non-pathogenic. In this study, a bacterial strain designated JMAV14 was isolated from the liver tissue of a diseased and deceased bovine in Chaoyang City, Liaoning Province, China. Phylogenetic analysis based on core genome single-copy orthologous genes and whole-genome SNP loci, combined with average nucleotide identity (ANI) analysis, identified JMAV14 at the species level of *Aeromonas salmonicida*. Further bacteriological identification methods, including VapA gene detection and physiological and biochemical characteristic tests, ultimately classified it as an atypical bovine-derived *Aeromonas salmonicida* subsp. *pectinolytica* strain. Pathogenicity assays showed that this strain caused a mortality rate of 70% in mice with a median lethal dose (LD₅₀) of 1.330 × 10^7^ CFU per mouse, and a mortality rate of 50% in crucian carp (*Carassius carassius*). These results indicate that JMAV14 possesses strong pathogenicity and broad cross-species infection ability, which challenges the traditional understanding that this subspecies is non-pathogenic. This study clarifies the biological characteristics of bovine-derived *Aeromonas salmonicida* subsp. *pectinolytica* during cross-species infection, providing a theoretical basis for the establishment of identification methods, diagnostic techniques, and prevention and control strategies for this bacterium.

## 1 Introduction

*Aeromonas salmonicida* is a Gram-negative, facultatively anaerobic bacterium with a short rod-like shape and rounded ends. It typically occurs in pairs or chains, lacks flagella but possesses numerous fine pili surrounding the cell, is non-spore-forming, and has a narrow capsule. Belonging to the class Gammaproteobacteria, order Aeromonadales, family Aeromonadaceae, and genus *Aeromonas*, this bacterium is non-motile. Currently, five subspecies of *Aeromonas salmonicida* are universally recognized: *Aeromonas salmonicida* subsp. *salmonicida*, *Aeromonas salmonicida* subsp. *achromogenes*, *Aeromonas salmonicida* subsp. *masoucida*, *Aeromonas salmonicida* subsp. *smithia*, and *Aeromonas salmonicida* subsp. *pectinolytica* (Liu, 2017). As a globally distributed and highly transmissible opportunistic pathogen, *A. salmonicida* can be transmitted through skin, gills, mouth, and blood (Wang et al., 2018), primarily causing diseases in fish of the families Salmonidae and Cyprinidae (Cao et al., 2009; Wang and Wang, 2015).

Relevant studies have shown that the host range of *Aeromonas salmonicida* is gradually expanding. In addition to infecting aquatic organisms and causing furunculosis, it can also infect humans and other mammals. Deng et al. (2019) first isolated *Aeromonas salmonicida* from the lungs, liver, and spleen of diseased pigs in a pig farm in Henan Province. Lin et al. (2011) detected *Aeromonas salmonicida* in the blood of goats in Fengdu County, Chongqing Municipality. Varshney et al. (2017) reported the first case of postoperative endophthalmitis caused by *Aeromonas salmonicida* in a cataract patient. These studies collectively demonstrate that *Aeromonas salmonicida* can infect fish, pigs, goats, and humans. However, there are no reports of *Aeromonas salmonicida* infections originating from cattle to date. In this study, we isolated and identified pathogens from diseased cattle in Chaoyang, Liaoning Province, performed whole-genome sequencing analysis, and investigated biological characteristics such as pathogenicity. This research is of great significance for enriching the theoretical understanding of the biological traits of cattle-derived *Aeromonas salmonicida* and its cross-species infection mechanisms. Additionally, it provides guidance for the identification of *Aeromonas salmonicida* subsp. *pectinolytica* and the establishment of corresponding diagnostic, prevention, and control measures.

## 2 Materials and methods

### 2.1 Sources of disease material

In April 2024, an adult bovine from a cattle farm in Chaoyang, Liaoning Province, China, developed illness and died. The clinical manifestations mainly included emaciation, elevated body temperature, and dyspnea. Postmortem examination revealed pathological lesions such as pulmonary congestion, uneven coloration, partial fleshy consolidation of the lungs, as well as hepatomegaly, uneven coloration, and fragile texture of the liver. Under sterile conditions, pathological samples including the liver and lungs were collected from the deceased bovine and stored at the Liaoning Provincial Key Laboratory of Animal Product Quality and Safety, Jinzhou Medical University. This study was conducted at the Liaoning Provincial Key Laboratory of Animal Product Quality and Safety, Jinzhou Medical University, from April 2024 to April 2025, with a total duration of 12 months.

### 2.2 Experimental animals

A total of 200 specific pathogen-free (SPF) Kunming mice (6 weeks old, weighing 18–22 g, half male and half female) were purchased from the Experimental Animal Center of Jinzhou Medical University. Sixty healthy crucian carp (*Carassius carassius*) (body length: 15 ± 1 cm, body weight: 35 ± 5 g) were also obtained from the same center and acclimated in an aquaculture system for 7 days prior to the experiment. This study protocol was approved by the Ethics Committee of Jinzhou Medical University (Approval No.: 20240126-25). All animal experiments were performed in strict accordance with the ethical guidelines formulated by the Ethics Committee of Jinzhou Medical University.

### 2.3 Bacterial isolation, purification, and morphological observation

Pathological samples including the liver and lungs were collected from the deceased bovine. Using a sterile inoculating loop, the lesion tissues were punctured, and the bacteria were streaked onto 10% sheep blood nutrient agar medium (TX0030, Solarbio, Beijing, China; HB0109, Hopebio, Qingdao, China) for isolation and culture at 37 °C for 18–24 h under constant temperature. Single colonies on the streaked plates were picked and subcultured. A single colony was subjected to Gram staining (HB8278, Hopebio, Qingdao, China), and bacterial morphology was observed under a microscope. After confirming a pure culture, the isolate was inoculated into Strain Store Medium (HBPT001-1, Hopebio, Qingdao, China), clearly labeled, and stored at −80 °C.

### 2.4 Biochemical test identification

In accordance with *Bergey’s Manual of Determinative Bacteriology* (Liao and Yen-Hsiung, 1992), the method described by Pavan et al. (2000), and the instructions of the bacterial micro-biochemical reaction tubes, the third-generation JMAV14 strain was resuscitated and inoculated into different bacterial micro-biochemical reaction tubes (SN068, GS005, GS006, HB8279, GS001, HB8281, GS003, GB007, GB101; Hopebio, Qingdao, China) respectively. Meanwhile, negative and positive control groups were set up. All tubes were incubated at 37 °C under constant temperature for the specified time, and the experimental results were observed and recorded.

### 2.5 16SrRNA sequence analysis

The genomic DNA of strain JMAV14 was extracted using a bacterial genomic DNA extraction kit (DP302-02, TIANGEN, Beijing, China). PCR amplification was performed with bacterial 16S rRNA primers (5′-AGAGTTTGATCATGGCTCAG-3′/5′-TACGGTTACCTTGTTACGACTT-3′). The PCR cycling conditions were as follows: initial denaturation at 95 °C for 5 min; followed by 30 cycles of denaturation at 95 °C for 45 s, annealing at 55 °C for 45 s, and extension at 72 °C for 45 s; a final extension at 72 °C for 10 min; and storage at 4 °C. After electrophoresis of the PCR amplicons on a 1.5% agarose gel (A620014, Sangon Biotech, Shanghai, China), the band size was observed using a gel imaging system (Tanon, Shanghai, China). Amplicons with the expected target band size were sent to Sangon Biotech (Shanghai) Co., Ltd. for sequencing. The 16S rRNA gene sequencing product of strain JMAV14 (approximately 1,500 bp in length) was subjected to homologous sequence alignment with the NCBI GenBank database using BLASTn. The parameter settings were expected value (e-value) ≤ 1e−5 and sequence similarity (percentage identity) ≥ 95%, and 30 closely related reference sequences were selected. After integrating the JMAV14 sequence with the aforementioned reference sequences, multiple sequence alignment was performed using DNAMAN software (v9.0) with default parameters. Following alignment, the sequence file was imported into MEGA 7.0 software, and a phylogenetic tree was constructed using the Neighbor-Joining (NJ) method. The reliability of the tree topology was evaluated by 1,000 bootstrap replicates.

### 2.6 VapA gene identification

Genomic DNA extraction was performed as described in Section 2.5. Referring to the VapA gene primers and experimental protocol reported by Wang et al. (2022), PCR amplification was conducted using the following VapA gene primers: 5′-ACAGTGCACCGAAGGTTGAT-3′ (forward) and 5′-ACGGCAGAGCTTGTCTACCT-3′ (reverse). The PCR cycling conditions were as follows: initial denaturation at 95 °C for 5 min; followed by 30 cycles of denaturation at 95 °C for 45 s, annealing at 55.7 °C for 45 s, and extension at 72 °C for 45 s; a final extension at 72 °C for 10 min; and storage at 4 °C. After electrophoresis of the PCR amplicons on a 1.5% agarose gel, the band size was visualized using a gel imaging system. Due to the lack of available vapA-positive *Aeromonas salmonicid* strains in domestic laboratories and restrictions on the import of pathogenic bacteria, no positive control for *Aeromonas salmonicida* was included in this experiment. However, the presence of the vapA gene in the JMAV14 genome was verified by whole-genome BLASTn analysis (reference sequence: GenBank accession no. MK244294.1).

### 2.7 Whole genome sequencing and analysis of bacteria

#### 2.7.1 Whole genome sequencing and gene splicing

The purified bacterial culture was sent to Sangon Biotech (Shanghai) Co., Ltd. for sequencing using the PacBio third-generation sequencing platform and Illumina second-generation sequencing platform. PacBio single-molecule sequencing data were assembled with Canu (v1.3) under default parameters (minReadLength = 1,000, minOverlapLength = 500). Illumina sequencing data were incorporated to fill gaps in the assembled contigs using GapFiller (v1.11) with default parameters (max_iterations = 100, min_overlap = 30). Sequence correction was performed using Pilon (v1.24) with the following parameters: --fixbases --fixindels --fixmates. The complete genome sequence of JMAV14 has been deposited in the NCBI GenBank database under the accession number CP191580 (only the genome of strain JMAV14 was submitted).

#### 2.7.2 Gene element analysis and advanced genome annotation

Gene elements including CDS, tRNA, and rRNA were predicted using NCBI-PGAP software (v1.2.1) with default parameters. CRISPR prediction analysis was performed via the CRT tool (v1.2) under default parameters (minimum repeat length = 24 bp, maximum repeat length = 47 bp). Genomic island prediction was conducted using IslandPath-DIMOB software (v0.2) with default parameters. Prophage prediction analysis was carried out using PhiSpy software (v2.3) with default parameters. The sequences of JMAV14 were aligned against the VFDB and CARD databases using Blastp software (v2.15.0) with the following parameters: e-value ≤ 1e−5, pident ≥ 40, and minlength ≥ 50, to obtain functional annotation information of virulence factors and antimicrobial resistance (AMR) genes.

#### 2.7.3 Gene function annotation

The sequences of JMAV14 were aligned against the NR, KEGG, and COG databases using DIAMOND software (v2.1.9) with the parameter: e-value ≤ 1e−5, to obtain annotation information. Additionally, sequence alignment against the GO database was performed using eggNOG-mapper software (v2.1.10) with default parameters to acquire corresponding annotation information.

#### 2.7.4 Comparative genomic analyses

The assembled genome sequence of JMAV14 was aligned against the NCBI nt database using the blastn program in NCBI BLAST+ (v2.2.28). Homologous strains were selected based on sequence bit-score, and a total of 29 representative reference strains with complete annotations in the database before August 9, 2024, were collected. Notably, the type strain of *Aeromonas salmonicida* subsp. *pectinolytica* (strain 34mel) was excluded as it failed to meet the screening criteria due to incomplete genome annotation and a bit-score below the threshold. Average Nucleotide Identity (ANI) analysis was performed using the ANIb method (based on blastn) implemented in JSpecies software (v1.2.1), with a species delineation threshold set at ANI ≥ 96% to complete the species identification at the genomic level.

#### 2.7.5 Construction of phylogenetic trees

The strains used in this study were the same as those described in Section 2.7.4. Two genome-based approaches were employed for phylogenetic analysis, namely the construction of a core genome tree and a whole-genome SNP tree, with the detailed procedures for each approach outlined below. For the core genome tree construction: single-copy orthologous genes among homologous strains were identified using OrthoFinder v2.5.4. The nucleotide sequences of these single-copy genes were concatenated, aligned using MAFFT v7.487 with the parameters --auto --maxiterate 1,000, and trimmed to remove regions with low alignment quality. A core genome phylogenetic tree was then constructed via the neighbor-joining (NJ) method implemented in FastTree v2.1.11 under the GTR+CAT model, with 1,000 bootstrap replicates performed to evaluate clade support values. For the whole-genome SNP tree construction: genome-wide SNP loci were detected using Snippy v4.6.0. High-quality, non-recombinant SNP loci were filtered with the following criteria: coverage depth ≥10×, allele frequency ≥90%, and exclusion of repetitive and recombinant regions. The filtered SNP sequences were aligned using MAFFT v7.487, followed by the construction of an SNP-based phylogenetic tree via the NJ method in FastTree v2.1.11 with 1,000 bootstrap replicates. The clade support values of both phylogenetic trees were expressed as bootstrap percentage (BP). Visualization and annotation of the trees were performed using the ggtree package (v3.4.4) in R v4.2.2.

### 2.8 Mice pathogenicity and LD_50_ test

#### 2.8.1 Pathogenicity tests

The mouse pathogenicity assay was designed with reference to Deng et al. (2019). Mice were divided into three groups: the infection group, negative control group, and blank control group, with 10 mice per group. For the infection group: bacterial cultures were grown at 37 °C, and each mouse was intragastrically administered 0.4 mL of bacterial suspension (concentration: 3.326 × 10^8^ CFU/mL, actual infection dose: 1.330 × 10^8^ CFU per mouse). For the negative control group: each mouse was intragastrically given 0.4 mL of sterile physiological saline. The blank control group received no treatment. Rationale for selecting the intragastric administration route: this route mimics the process of bacterial invasion through the digestive tract in natural infections, which is consistent with the potential transmission mode of *Aeromonas salmonicida* via food or water sources. Survival curves were plotted using the Kaplan–Meier method, and survival differences between the infection group and control groups were compared by the Log-rank (Mantel-Cox) test. Differences in mortality rates between the infection group and control groups were analyzed using the Chi-square test. Statistical analyses were performed with SPSS 26.0 software, and survival curves were generated using GraphPad Prism 9.0. The statistical significance level was set at *p* < 0.05.

#### 2.8.2 LD_50_ test

The infection group was divided into five subgroups, with 10 mice per subgroup. Each mouse was intragastrically administered 0.4 mL of bacterial suspension at different concentrations: 3.326 × 10^9^ CFU/mL (actual infection dose: 1.330 × 10^9^ CFU per mouse), 3.326 × 10^8^ CFU/mL (actual infection dose: 1.330 × 10^8^ CFU per mouse), 3.326 × 10^7^ CFU/mL (actual infection dose: 1.330 × 10^7^ CFU per mouse), 3.326 × 10^6^ CFU/mL (actual infection dose: 1.330 × 10^6^ CFU per mouse), and 3.326 × 10^5^ CFU/mL (actual infection dose: 1.330 × 10^5^ CFU per mouse). The negative control group (10 mice) received 0.4 mL of sterile physiological saline via intragastric administration per mouse, while the blank control group (10 mice) received no treatment. The rationale for selecting the intragastric administration route was the same as described in Section 2.8.1. The median lethal dose (LD₅₀) was calculated using the Karber’s method, with the formula as follows Equation 1:


$$\mathrm{lg}(LD_{50})=X_{K}-d(\sum P_{i}-0.5)\;\;\;$$ (1)

$X_{K}$ represents the logarithm of the maximum dose; d denotes the difference between the logarithms of adjacent doses; $P_{i}$ is the mortality rate; and i is the subgroup number.

In the above mouse experiments, clinical symptoms and infection status of mice were observed continuously for 10 days. Deceased mice were subjected to postmortem examination to observe pathological changes. Pathological samples from affected organs of experimental mice were collected under sterile conditions, inoculated onto 10% sheep blood nutrient agar medium for isolation and culture, and subjected to bacteriological detection. At the end of the experiment, mice were euthanized in accordance with the guidelines of the American Veterinary Medical Association (AVMA). Euthanasia was performed by intraperitoneal injection of a lethal dose of sodium pentobarbital (300 mg/kg, Sigma-Aldrich, USA) followed by cervical dislocation.

Statistical analysis: data were processed using SPSS 26.0 software. Differences in mortality rates between the infection groups and control groups were analyzed by the Chi-square test, with *p* < 0.05 considered statistically significant. The LD₅₀ value and its 95% confidence interval (95% CI) were calculated using the Karber’s method with the confidence interval approach. Survival curves of different dose groups were constructed using the Kaplan–Meier method, and survival differences among multiple groups (5 infection subgroups + 1 control group) were analyzed by the Log-rank test. Based on the survival curves and mortality data of each group, the LD₅₀ and 95% CI were further verified using the Karber’s method. GraphPad Prism 9.0 was used for plotting, and the statistical significance level was set at *p* < 0.05.

#### 2.8.3 Detection of bacterial load in mouse organs

To avoid interfering with the natural observation of the mouse pathogenicity assay and median lethal dose (LD₅₀) test, a parallel replicate experimental design was adopted in this study. Separate parallel bacterial load groups were established with identical conditions to the aforementioned core experiments. Each group consisted of 10 mice, and the infection route, bacterial suspension concentration, and actual infection dose were consistent with those described in Section 2.8.1 (Pathogenicity Assay) and Section 2.8.2 (LD₅₀ Test) (0.4 mL of the corresponding concentration of bacterial suspension administered intragastrically).

At 24 h, 48 h, and 72 h post-infection, 3 mice were randomly selected from each group and euthanized in accordance with the guidelines of the American Veterinary Medical Association (AVMA) (intraperitoneal injection of 300 mg/kg sodium pentobarbital followed by cervical dislocation). Target organs including the liver, spleen, lungs, and kidneys were isolated under sterile conditions. Each organ tissue was homogenized into a 10% (w/v) suspension with sterile physiological saline. After serial dilution, 100 μL of the dilution was spread onto 10% sheep blood nutrient agar medium and incubated at 37 °C for 18–24 h. Colony counting was performed to calculate the bacterial load per gram of tissue (CFU/g tissue). The negative control group and blank control group underwent the same procedures simultaneously. The experiment included 3 biological replicates, and data were expressed as “mean ± standard deviation ( $\bar{x}\pm s$ ).” Statistical analysis was performed using SPSS 26.0 software, and intergroup differences were tested by one-way analysis of variance (ANOVA). A *p*-value <0.05 was considered statistically significant.

### 2.9 Histopathological observations in mice

Pathological tissue samples of the heart, liver, spleen, lungs, kidneys, brain, and duodenum were collected separately from deceased mice in the infection group and healthy mice in the control group. The samples were fixed in 10% formalin solution, and paraffin-embedded tissue sections were prepared following the standard hematoxylin–eosin (HE) staining protocol. Pathological changes in each organ tissue were observed and recorded using a microscopic image analysis system (Leica, Germany), with simultaneous image acquisition. The criteria for assessing pathological symptoms were referenced from *Veterinary Clinical Pathology Guidelines* (Freeman and Klenner, 2015).

### 2.10 Fish pathogenicity tests

Fish were divided into an infection group, a negative control group, and a blank control group, with 10 fish per group. For the infection group: bacterial cultures were incubated at 37 °C, and each fish was intramuscularly injected with 0.3 mL of bacterial suspension (concentration: 3.326 × 10^7^ CFU/mL, actual infection dose: 9.978 × 10^6^ CFU per fish). For the negative control group: each fish received an intramuscular injection of 0.3 mL of sterile physiological saline. The blank control group was not subjected to any treatment. Rationale for selecting the intramuscular injection route: Crucian carp have an intact body surface barrier; intramuscular injection enables precise control of the infection dose and directly mimics the natural infection scenario where bacteria invade through skin wounds. The experimental fish were reared under the following conditions: water temperature of 20.5–23 °C and pH of approximately 7.0. Clinical symptoms and infection status of the fish were observed continuously for 10 days. Deceased fish were subjected to postmortem examination to observe pathological changes. Pathological tissues were collected under sterile conditions as samples, inoculated onto 10% sheep blood nutrient agar medium, and subjected to bacterial isolation and culture for bacteriological detection. At the end of the experiment, fish were euthanized according to the recommendations of the Canadian Council on Animal Care (CCAC). Euthanasia was performed by immersing the fish in a 200 mg/L solution of tricaine methanesulfonate (MS-222) (pH adjusted to 7.0 with sodium bicarbonate) followed by decapitation.

### 2.11 Statistical analysis

Differences in mortality rates between the infection group and control groups were analyzed using the Chi-square test with SPSS 26.0 software, with *p* < 0.05 considered statistically significant. Survival curves were plotted using the Kaplan–Meier method, and survival differences between the infection group and control groups were compared via the Log-rank test. Statistical analyses were performed using SPSS 26.0 software, and survival curves were generated using GraphPad Prism 9.0. The statistical significance level was set at *p* < 0.05.

#### 2.11.1 Detection of bacterial load in fish organs

To avoid interfering with the observation of natural disease onset in the fish pathogenicity assay, a parallel replicate experimental design was adopted. Separate parallel bacterial load groups were established with identical conditions to those described in Section 2.10 (Fish Pathogenicity Assay). Each group consisted of 10 fish, and the infection route, bacterial suspension concentration, and actual infection dose were consistent with the core experiment: each fish was intramuscularly injected with 0.3 mL of bacterial suspension (concentration: 3.326 × 10^7^ CFU/mL, actual infection dose: 1.00 × 10^7^ CFU per fish). The negative control group was injected with an equal volume of sterile physiological saline, while the blank control group received no treatment. All experimental fish were reared in a standardized aquaculture environment with water temperature maintained at 20.5–23 °C and pH at approximately 7.0.

At 24 h, 48 h, and 72 h post-infection, 3 fish were randomly selected from each group and euthanized via overdose anesthesia with MS-222. Target organs (major immune and metabolic organs of fish), including the liver, kidneys, and gills, were rapidly isolated under sterile conditions. Each organ tissue was homogenized into a 10% (w/v) suspension using sterile physiological saline. After 10-fold serial dilution, 100 μL of the dilution was spread onto 10% sheep blood nutrient agar medium and incubated at 37 °C for 18–24 h. Colony counting was then performed to calculate the bacterial load per gram of tissue (CFU/g tissue). The negative control group and blank control group underwent the same procedures simultaneously. The experiment was conducted with 3 biological replicates, and data were expressed as “mean ± standard deviation ( $\bar{x}\pm s$ ).” Statistical analysis was performed using SPSS 26.0 software, and intergroup differences were tested by one-way analysis of variance (ANOVA). A *p*-value < 0.05 was considered statistically significant.

### 2.12 Antimicrobial susceptibility testing of strain JMAV14

The broth microdilution method was employed to determine the antimicrobial susceptibility of strain JMAV14. A total of 18 commonly used antimicrobial agents were selected, including ampicillin, amoxicillin, clavulanate potassium, ceftiofur, ceftazidime, meropenem, gentamicin, spectinomycin, apramycin, tetracycline, florfenicol, sulfisoxazole, trimethoprim, sulfamethoxazole, enrofloxacin, ofloxacin, mequindox, and colistin. The isolate was inoculated into 10% bovine serum albumin-Mueller Hinton (BSA-MH) broth and cultured to the logarithmic growth phase in a constant temperature shaking incubator at 37 °C with shaking at 160 rpm. The bacterial suspension concentration was adjusted to 0.5 McFarland standard (equivalent to 1.0 × 10^8^ CFU/mL) using a McFarland nephelometer. Subsequently, 60 μL of the adjusted bacterial suspension was added to 12 mL of 10% bovine serum albumin-Mueller-Hinton broth. A volume of 100 μL of the inoculum was dispensed into each well of a susceptibility testing plate for aerobic Gram-negative bacteria, followed by incubation at 37 °C in the dark for 17 h.

The results of the antimicrobial susceptibility test were interpreted primarily with reference to the *Aeromonas*-specific interpretive criteria outlined in CLSI M45-A3 [Clinical and Laboratory Standards Institute (CLSI), 2015]. Among the tested antimicrobials, 5 agents (including ceftazidime and meropenem) were eligible for interpretation using this dedicated standard. For the remaining antimicrobials (e.g., ampicillin, spectinomycin) without corresponding criteria in CLSI M45-A3, interpretation was further performed based on the general interpretive standards for aerobic Gram-negative bacteria specified in the *Clinical and Laboratory Standards Institute (CLSI) Performance Standards for Antimicrobial Susceptibility Testing* (M100-Ed34, 2024) (Clinical and Laboratory Standards Institute, 2024). Notably, these general standards have not been validated for *Aeromonas salmonicida* and are provided for *in vitro* reference only.

## 3 Results

### 3.1 Results of bacterial isolation, culture, morphological observation, and biochemical test identification

**Figure 1:**
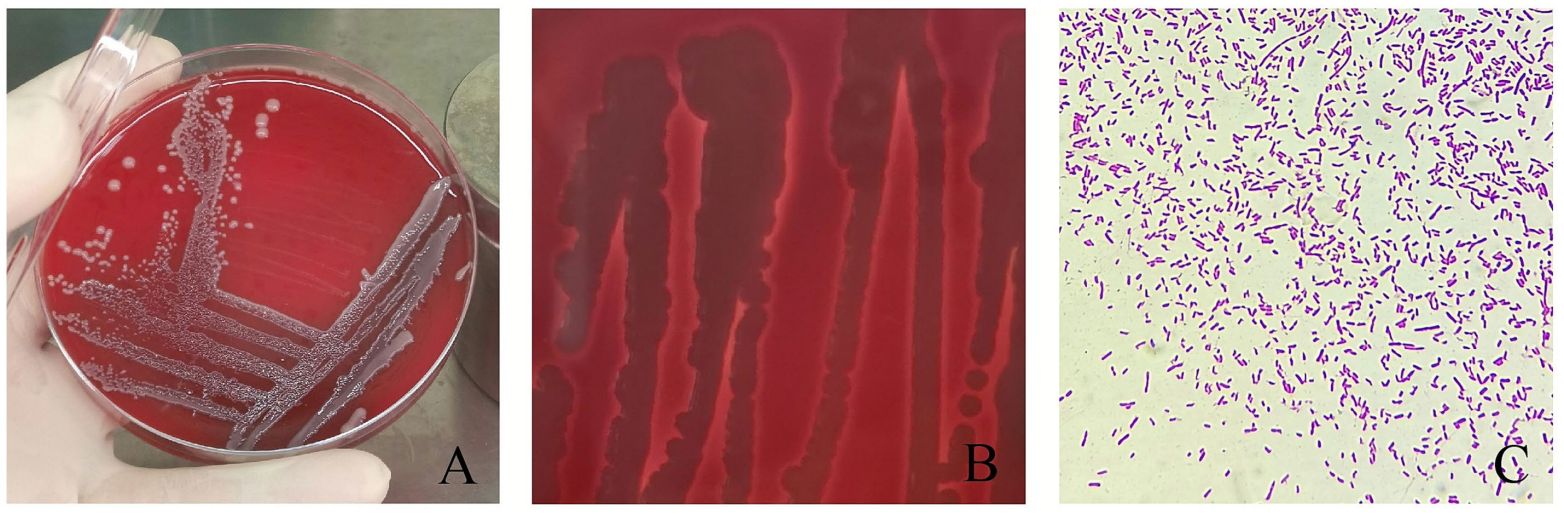
Growth morphology of JMAV14. **(A)** 10% sheep blood nutrient agar medium, **(B)** β haemolysis, **(C)** Gram stain, microscopic observation (100×).

Colonies of JMAV14 grown on 10% sheep blood nutrient agar were round, smooth, and white, with β-hemolysis (Figures 1A,B). Microscopic examination of Gram-stained smears revealed Gram-negative, red, short rod-shaped bacteria (Figure 1C), which were consistent with the cultural and morphological characteristics of *Aeromonas salmonicida*.

Biochemical test results showed that JMAV14 was able to grow at 37 °C. It tested positive for sucrose fermentation, brown diffusible pigment production, indole production, Voges-Proskauer (VP) test, mannitol fermentation, and glucose gas production, while the lysine decarboxylase test yielded a negative result (Table 1). These findings were consistent with the biochemical characteristics of *Aeromonas salmonicida* subsp. *pectinolytica*.

**Table 1:** Biochemical test results.

Test	*Aeromonas salmonicida* subspecies					
	JMAV14	*Pectinolytica*	*Achromogenes*	*Masoncida*	*Salmonicida*	*Smithia*
Sucrose fermentation	+	+	+	+	+	+
LDC	−	−	v	v	v	v
Brown diffusible pigment	+	+	−	−	+	−
Growth at 35–37 °C	+	+	−	−	−	−
Indole	+	+	+	+	−	+
Voges–Proskauer at 25 °C	+	+	−	+	−	−
Mannitol fermentation	+	+	−	+	+	−
Gas from glucose	+	+	−	+	−	ND

+, >85% positive; −, <15% positive; V, variable (15 ± 85%); ND, nodata.

### 3.2 Results of 16SrRNA gene identification

**Figure 2:**
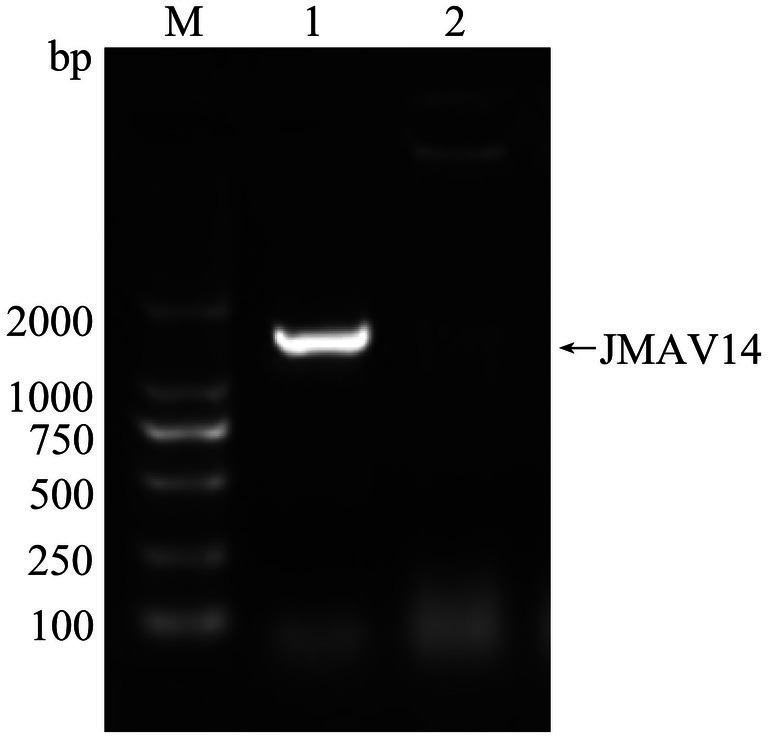
16S rRNA primer amplification plot. M: DM2000Marker, 1: test sample, 2: negative control.

**Figure 3:**
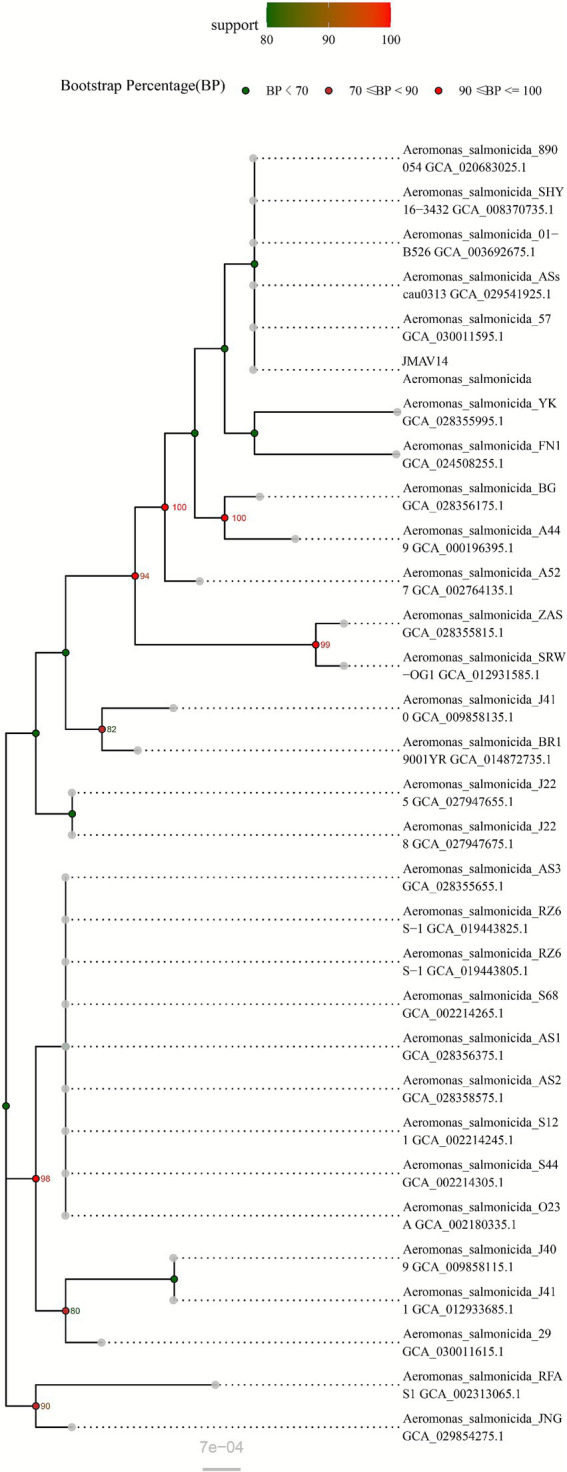
Neighbor-joining (NJ) phylogenetic tree of *Aeromonas salmonicida* strains based on 16S rRNA sequences.

Following PCR amplification and electrophoresis of the 16S rRNA gene from the JMAV14 DNA template, a target band of 1,500 bp in size was obtained (Figure 2). After sequencing of the amplicons, a 16S rRNA gene phylogenetic tree was constructed using MEGA 7.0 software via the Neighbor-Joining method with 1,000 bootstrap replicates. The results showed that JMAV14 clustered in the same clade as known strains of *Aeromonas salmonicida* in the phylogenetic tree. Combined with the result of sequence homology ≥ 99%, JMAV14 was identified as belonging to *Aeromonas salmonicida*. It should be noted that the bootstrap support value of the clade containing JMAV14 was <70, indicating that the detailed phylogenetic relationship between JMAV14 and its closely related strains needs to be further verified by whole-genome data (Figure 3).

The tree was constructed using the Neighbor-Joining (NJ) method with 1,000 bootstrap replicates. The colors of the dots on the branches represent bootstrap support values (BP): green (BP < 70), orange (70 ≤ BP < 90), and red (90 ≤ BP ≤ 100). The scale bar of “7e−4” indicates the sequence divergence corresponding to branch lengths (unit: nucleotide substitution rate).

### 3.3 Results of VapA gene identification

**Figure 4:**
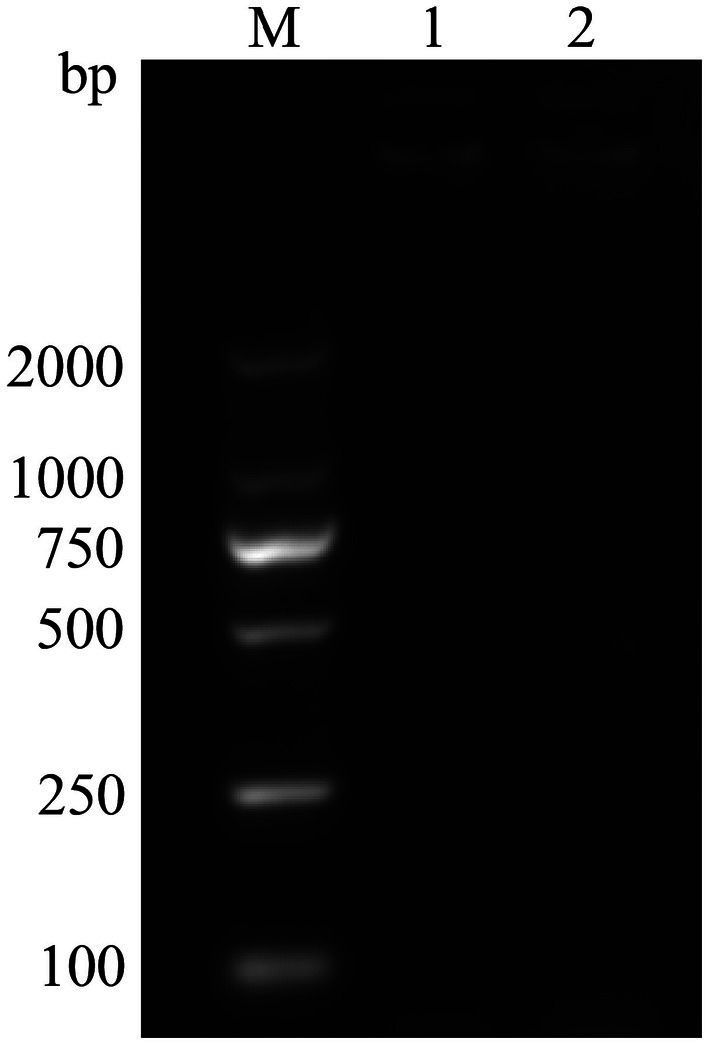
VapA primer amplification plot. M: DM2000Marker, 1: test sample, 2: negative control.

Following PCR amplification and electrophoresis of the *vapA* gene from the JMAV14 DNA template, no target band was detected (Figure 4). This result was consistent with the characteristic that *Aeromonas salmonicida* subsp. *pectinolytica* does not harbor the *vapA* gene, confirming that JMAV14 belongs to *Aeromonas salmonicida* subsp. *pectinolytica*.

### 3.4 Genome assembly and quality evaluation of JMAV14

Whole-genome sequencing of JMAV14 generated 6.8 Gb of raw Illumina data and 5.2 Gb of raw PacBio data, with an average sequencing depth of 172.63×. The assembly results showed that the genome had a total length of 4,818,586 bp, consisting of only one contig (chromosome-level assembly). The N50 length was identical to the total genome length (4,818,586 bp), and the GC content was 59.0%. BUSCO assessment indicated that the genome contained 124 complete BUSCO core genes, with no missing or fragmented genes. CheckM analysis yielded a genome completeness of 100% and a contamination rate of 0%. These results demonstrated that the genome assembly was of high quality with no significant contamination, which was sufficient to meet the requirements of subsequent analyses (Table 2).

**Table 2:** Genome assembly statistics of strain JMAV14.

Indicator	Value
Genome length (bp)	4,818,586
Number of contigs	1
N50 length (bp)	4,818,586
GC content (%)	58.6
Number of coding genes	4,326
BUSCO completeness (%)	100
CheckM completeness (%)	100
CheckM contamination (%)	0
Average sequencing depth (×)	172.63
Q20 ratio (%)	99.20
Q30 ratio (%)	97.67

### 3.5 Gene element analysis and advanced genome annotation of JMAV14

**Figure 5:**
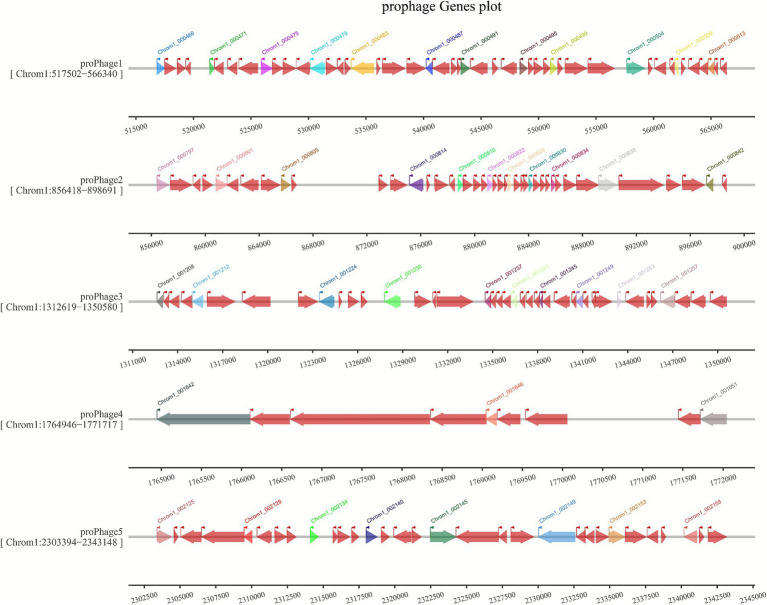
Genetic structure map of prophages. Each row represents one prophage fragment predicted by bioinformatics tools (labeled as ProPhage 1–ProPhage 5), with the genomic location interval of each prophage shown in parentheses [e.g., (Chrom1:517502–566,540)]. Each arrow represents a single gene, and the direction of the arrow corresponds to the transcriptional direction of the gene. Arrows of different colors represent genes with different functional categories, and the gene ID (e.g., Smart_05881) is labeled above each corresponding gene. The scale bar at the bottom of the figure indicates the genomic base positions, which is used to locate the distribution intervals of prophages and their genes.

**Figure 6:**
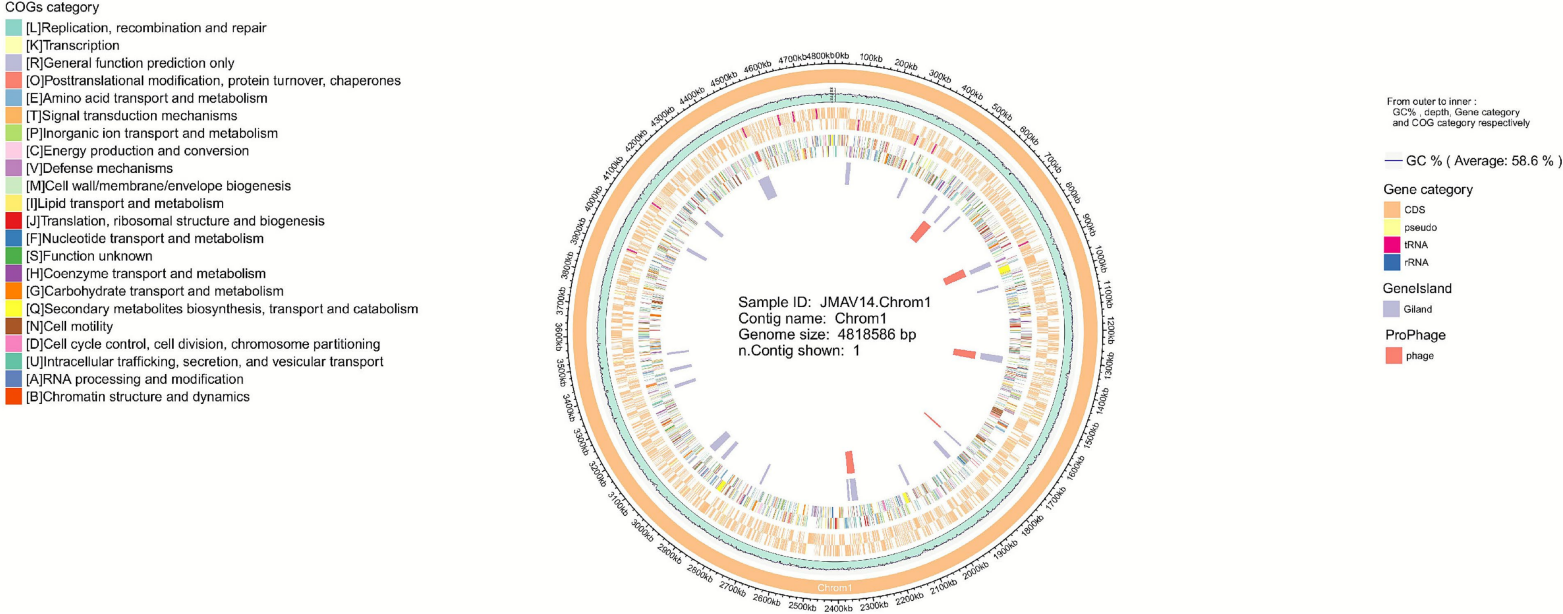
Genomic Circos plot. From the innermost to the outermost circle, the plot sequentially displays the following elements: prophages (light red circle), genomic islands (light purple circle), COG functional classification (colored circle), core genetic elements (light orange: coding sequences; yellow: pseudogenes; blue: ribosomal RNA; red: transfer RNA), GC content (light green circle), and contig distribution with corresponding IDs (orange circle).

Whole-genome sequencing results revealed that the JMAV14 genome has a total length of 4,818,586 bp with a G+C content of 58.6%. A total of 4,186 coding sequences (CDSs) were predicted, including 11 copies of 5S rRNA, 10 copies of 16S rRNA, 10 copies of 23S rRNA, and 124 copies of tRNA. The genome harbors 22 genomic islands and 5 intact prophages (Figure 5), with their boundary ranges as follows: 517,502–566,340 bp (Prophage 1), 856,418–898,691 bp (Prophage 2), etc. Each prophage contains 18–32 genes, mainly involving phage structural proteins and replication-related genes (see Supplementary Table 1: detailed annotation of prophage genes). No CRISPR repeats or spacer sequences were detected in the genome, indicating that this strain lacks the CRISPR-Cas immune system (Table 3). Virulence annotation of prophage genes showed no sequences with high homology (≥80% identity) to known virulence factors in the VFDB database, and the positional association between prophages and virulence-related genes remains unclear. A circular genomic map of JMAV14 was generated based on the predicted genomic information (Figure 6).

**Table 3:** Genomic information of strain JMAV14.

Item	Result
Genome size/bp	4,818,586
CDS/number	4,186
rRNA/number	31
tRNA/number	124
GC content/%	58.6
Genomic islands/number	22
Prophages/number	5
CRISPR sequences/number	0

Annotation of JMAV14 virulence factor gene sequences against the Virulence Factor Database (VFDB) showed that a total of 681 virulence genes were identified in VFDB SetB (predicted virulence genes). From these, 449 virulence genes belonging to VFDB SetA (experimentally validated virulence genes) were screened out. After manual verification with a homology threshold of ≥ 80%, 80 core virulence genes were finally retained, which could be classified into 6 categories of core functions: Motility and chemotaxis-related factors (28 genes, 35%): Including flagellar motor switch protein FliM and chemotaxis protein CheW-2. These factors regulate the flagellar motility and chemotaxis of the strain, helping JMAV14 locate host cells and break through mucosal barriers, thus laying the foundation for subsequent colonization. Type IV pilus assembly-related factors (22 genes, 27.5%): Containing pilus assembly ATPase TapB and pilus structural protein TppC. Type IV pili are core structures for bacterial adhesion to host surfaces, and the high proportion of these factors suggests that JMAV14 has a strong host colonization ability. Toxin secretion system-related factors (15 genes, 18.75%): Covering hemolysin transporter HlyB and RTX toxin-related protein RtxE. These factors are responsible for transporting virulence effector molecules such as hemolysins into host cells, disrupting cell membrane integrity and inducing tissue damage, which are direct pathogenic factors of JMAV14. Iron uptake-related factors (8 genes, 10%): Such as siderophore receptor FepA and iron-binding protein FbpB. These factors enable the strain to efficiently acquire iron ions in the iron-restricted host environment to meet the demands of growth and reproduction in the host. Biofilm formation-related factors (4 genes, 5%): Including alginate synthase AlgC and biofilm regulatory protein IcaA. These factors can promote biofilm formation by JMAV14, enhancing its resistance to host immune clearance and antibiotics. Metabolism and regulation-related factors (3 genes, 3.75%): Such as stringent response regulator RelA and phosphate transfer protein PhoP. These factors maintain the survival stability of the strain in the host by regulating metabolic adaptation and stress responses (see Supplementary Table 2: core virulence genes).

Results of annotation against the Comprehensive Antibiotic Resistance Database (CARD) showed that a total of 229 antibiotic resistance-related genes were identified in JMAV14. After manual verification with a homology threshold of ≥70%, 12 core resistance genes were finally retained. The resistance mechanism of JMAV14 exhibited the characteristics of *“antibiotic inactivation as the dominant mechanism combined with multi-mechanism synergy,”* with the specific distribution of the four resistance mechanisms as follows: Antibiotic inactivation (6 genes, 50.0%): As the core resistance mechanism, it includes *catB3* (chloramphenicol), *AAC (6′)-Ib4* (aminoglycosides), *aadA10* (aminoglycosides), *imiS* (carbapenems), *OXA-917* (carbapenems + cephalosporins + penicillins), and *MOX-9* (cephalosporins + cephamycins + penicillins). All these genes exert resistance by encoding enzymes that degrade antibiotics via enzymatic reactions. Antibiotic efflux (3 genes, 25.0%): Containing *qacEdelta1* (disinfectants and preservatives), *CRP* (fluoroquinolones + macrolides + penicillins), and *rsmA* (diaminopyrimidines + fluoroquinolones + chloramphenicol). These genes expel antibiotics out of bacterial cells through efflux pumps such as the Major Facilitator Superfamily (MFS). Antibiotic target alteration (2 genes, 16.7%): including *bacA* and *ugd* genes, both targeting peptide antibiotics. They reduce the drug-binding capacity by altering the structure of drug targets. Antibiotic target replacement (1 gene, 8.3%): Only the *sul1* gene, which targets sulfonamides. It mediates resistance by encoding a resistant dihydropteroate synthase to replace the sensitive target (see Supplementary Table 3: Core Antimicrobial Resistance Genes).

Whole-genome BLASTn analysis confirmed that there was no *vapA* homologous sequence in the JMAV14 genome (identity = 42%, coverage = 30%), which was consistent with the negative result of *vapA* gene PCR amplification (no 400 bp target band detected; Figure 4). This double verification ruled out false-negative results and clearly confirmed that JMAV14 does not carry the *vapA* gene.

### 3.6 Gene functional annotation of JMAV14

**Figure 7:**
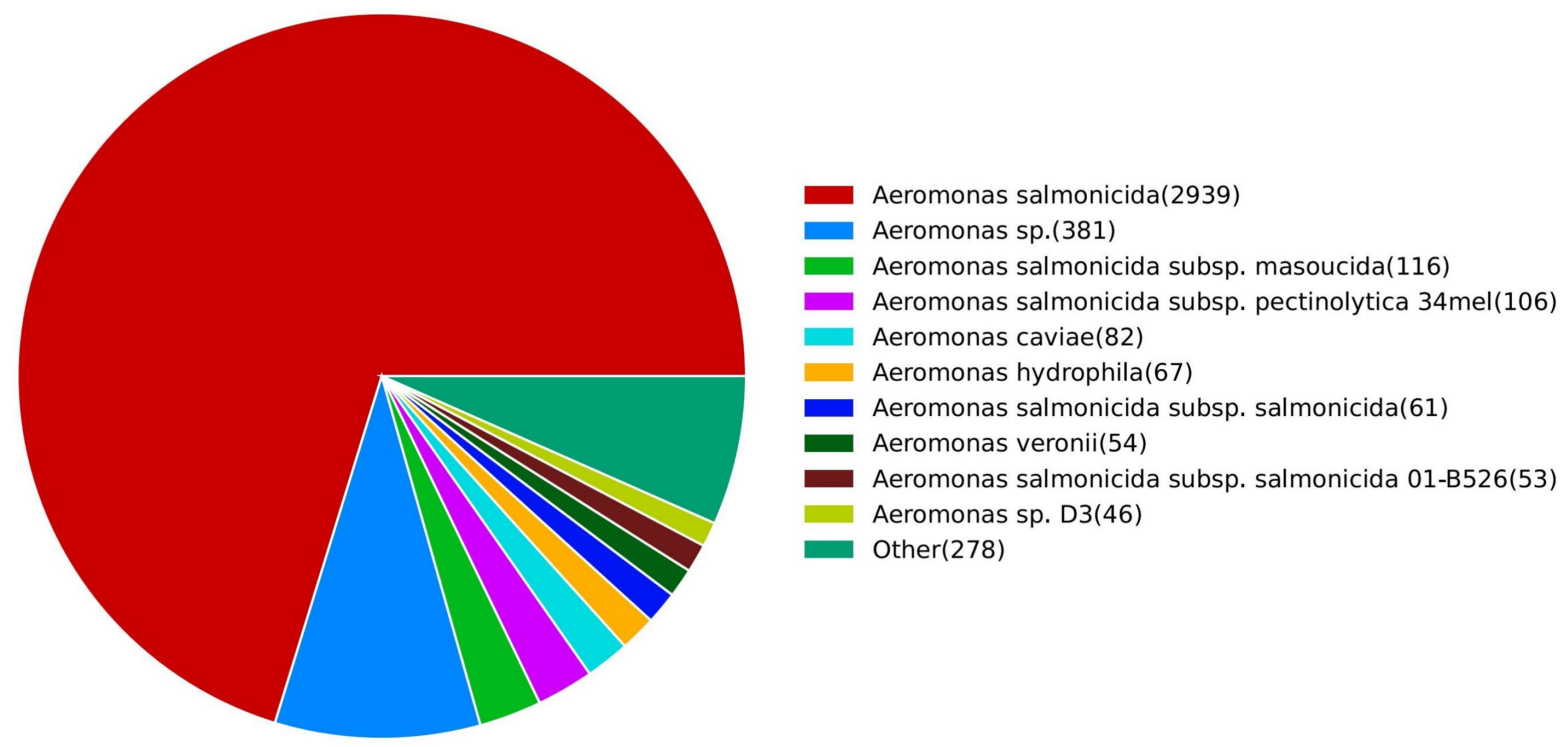
Pie chart of homologous species distribution. Each sector in the chart represents a single species. A larger sector area indicates a greater number of sequences aligned to that corresponding species.

In the NCBI non-redundant protein sequence database (NR), the annotated gene entries of JMAV14 showed the highest homology to those of *Aeromonas salmonicida*, accounting for 70.26% of the total annotated entries (Figure 7). This finding further verified the taxonomic affiliation of JMAV14 from the perspective of functional homology.

**Figure 8:**
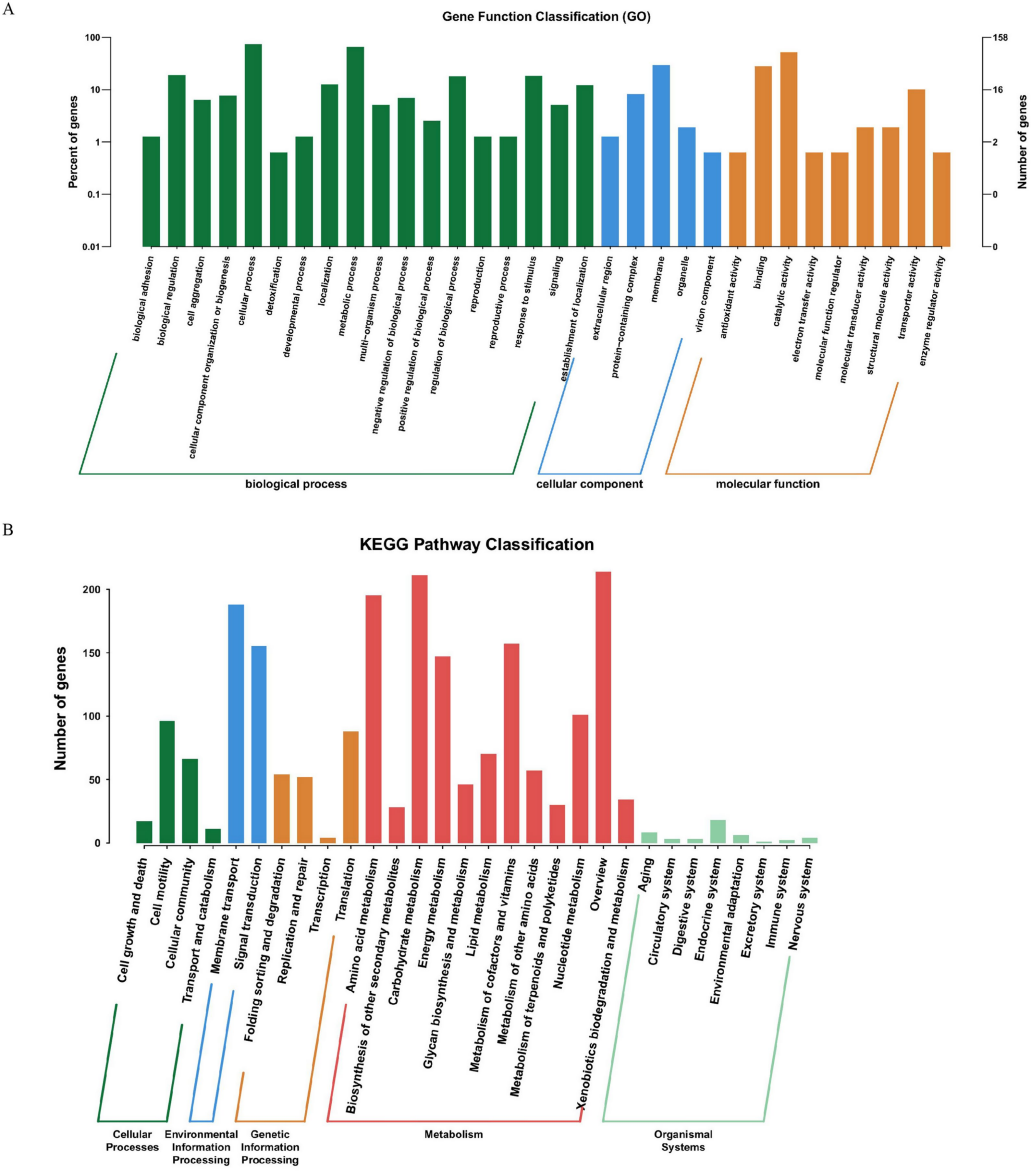
**(A)** GO annotation distribution bar chart; **(B)** KEGG pathway classification bar chart. **(A)** The abscissa represents the secondary classification of GO, and the ordinate represents the number of genes in the classification (right) and their percentage of the total annotated genes (left). Different colors indicate different orthologs. **(B)** The abscissa denotes the names of the involved metabolic pathways, and the ordinate represents the number of genes annotated to each pathway.

In the annotation against the Gene Ontology (GO) database (Figure 8A), entries classified under the biological process category accounted for the highest proportion (approximately 56.25%). The core functions were involved in cellular process, metabolic process, and biological regulation, indicating that JMAV14 possesses a complete set of regulatory capabilities for basic life activities, which provides functional support for its adaptation to the host environment and maintenance of pathogenicity.

In the Kyoto Encyclopedia of Genes and Genomes (KEGG) database annotation (Figure 8B), the Metabolism category contained the largest number of metabolic pathways. The core pathways included Overview (covering basic energy metabolic pathways such as glycolysis and the tricarboxylic acid cycle), Carbohydrate metabolism, and Amino acid metabolism. These results suggested that JMAV14 can meet the demands of growth and reproduction in different hosts through efficient substance and energy metabolism.

**Figure 9:**
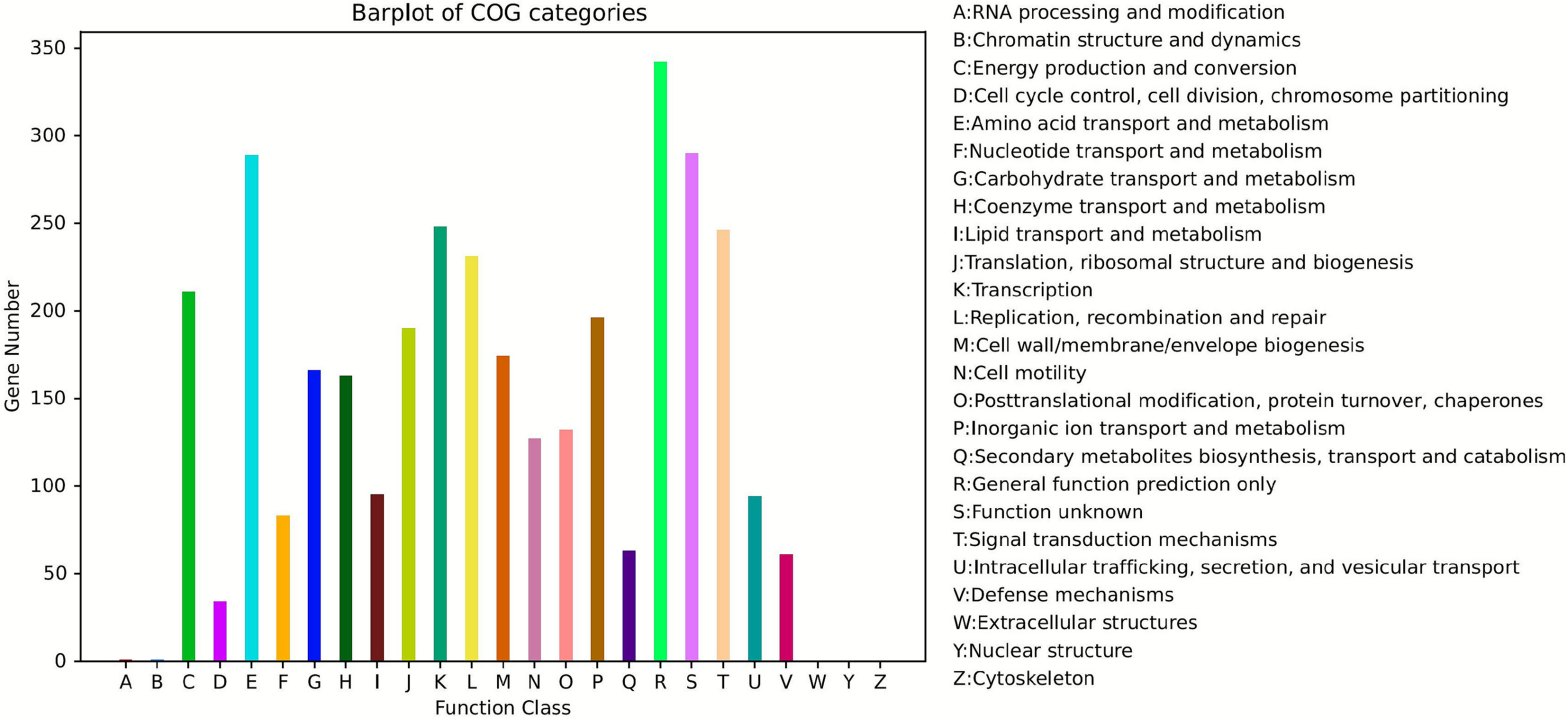
Bar chart of COG classification. Each color on the abscissa represents a functional category of COG, and the ordinate indicates the number of genes annotated to the corresponding category.

In the annotation against the Clusters of Orthologous Groups of proteins (COG) database (Figure 9), the top three functional categories with the largest number of genes were as follows: *general function prediction only* (342 genes), *function unknown* (290 genes), and *Amino acid transport and metabolism* (289 genes). Among these, the enrichment of genes related to amino acid transport and metabolism implied that JMAV14 may enhance its colonization and pathogenicity by regulating the uptake and utilization of amino acids in the host. Meanwhile, the presence of function-unknown genes provides a direction for further exploration of its unique pathogenicity-related functions.

### 3.7 ANI analysis results of JMAV14

**Figure 10:**
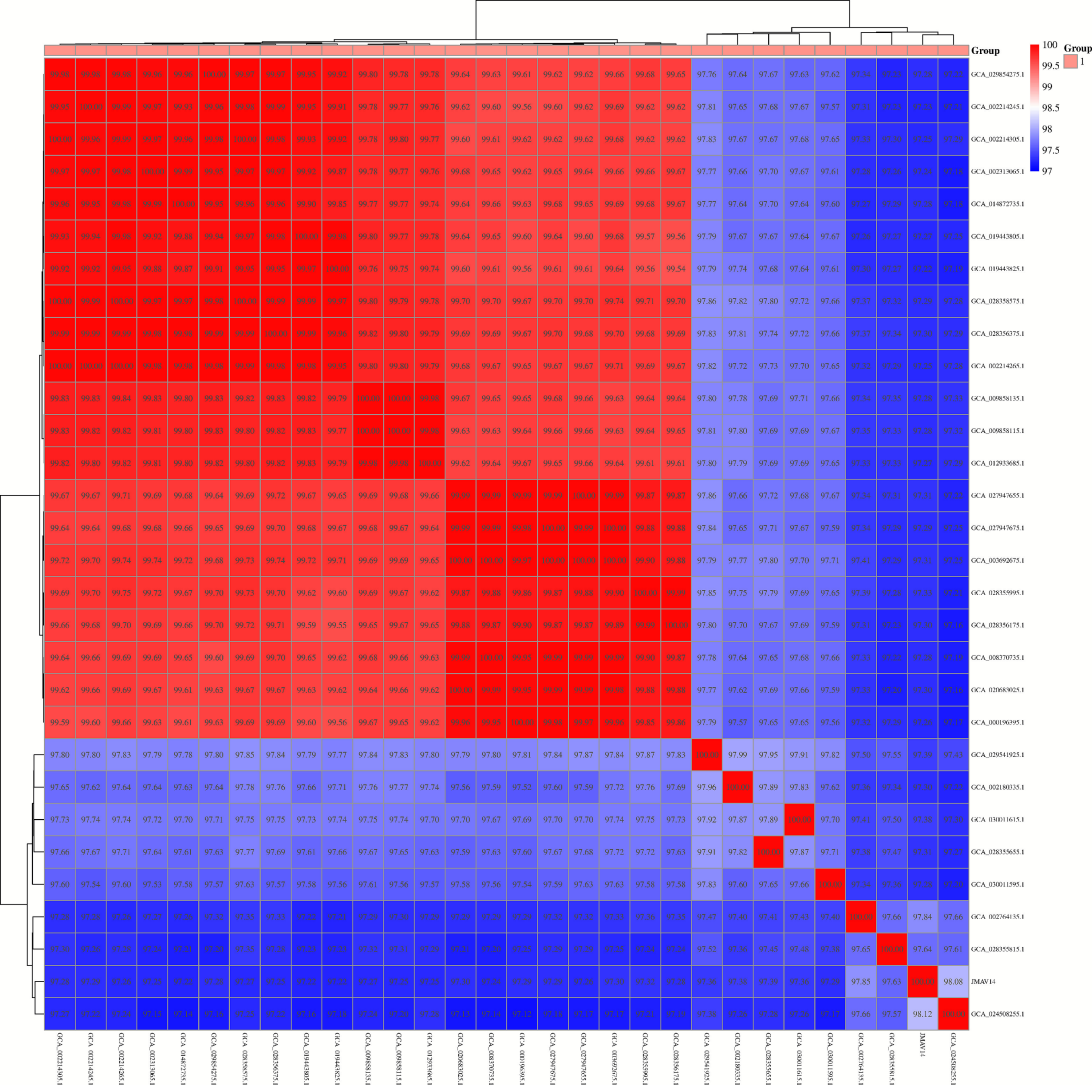
ANI-based bidirectional clustering heatmap.

The average nucleotide identity (ANI) analysis results of JMAV14 showed that it shared the highest ANI value (98.08%) with strain FN1 (GCA_024508255.1), which was above the species delineation threshold (≥96%). Strain FN1 is annotated as *Aeromonas salmonicida* in the NCBI database, thus confirming that JMAV14 belongs to *Aeromonas salmonicida* at the genomic level. In addition, the ANI values between JMAV14 and the 29 reference strains were all greater than 96%, which clearly reflects the whole-genome homology distribution of JMAV14 at the species level and further verifies the reliability of its taxonomic classification (Figure 10).

This heatmap illustrates the genetic similarity (average nucleotide identity, ANI) among strains, along with the results of bidirectional clustering (both rows and columns). The color gradient from red to blue represents a decrease in ANI values from high to low.

### 3.8 Phylogenetic trees of *Aeromonas salmonicida* based on single-copy gene sets and SNPs

**Figure 11:**
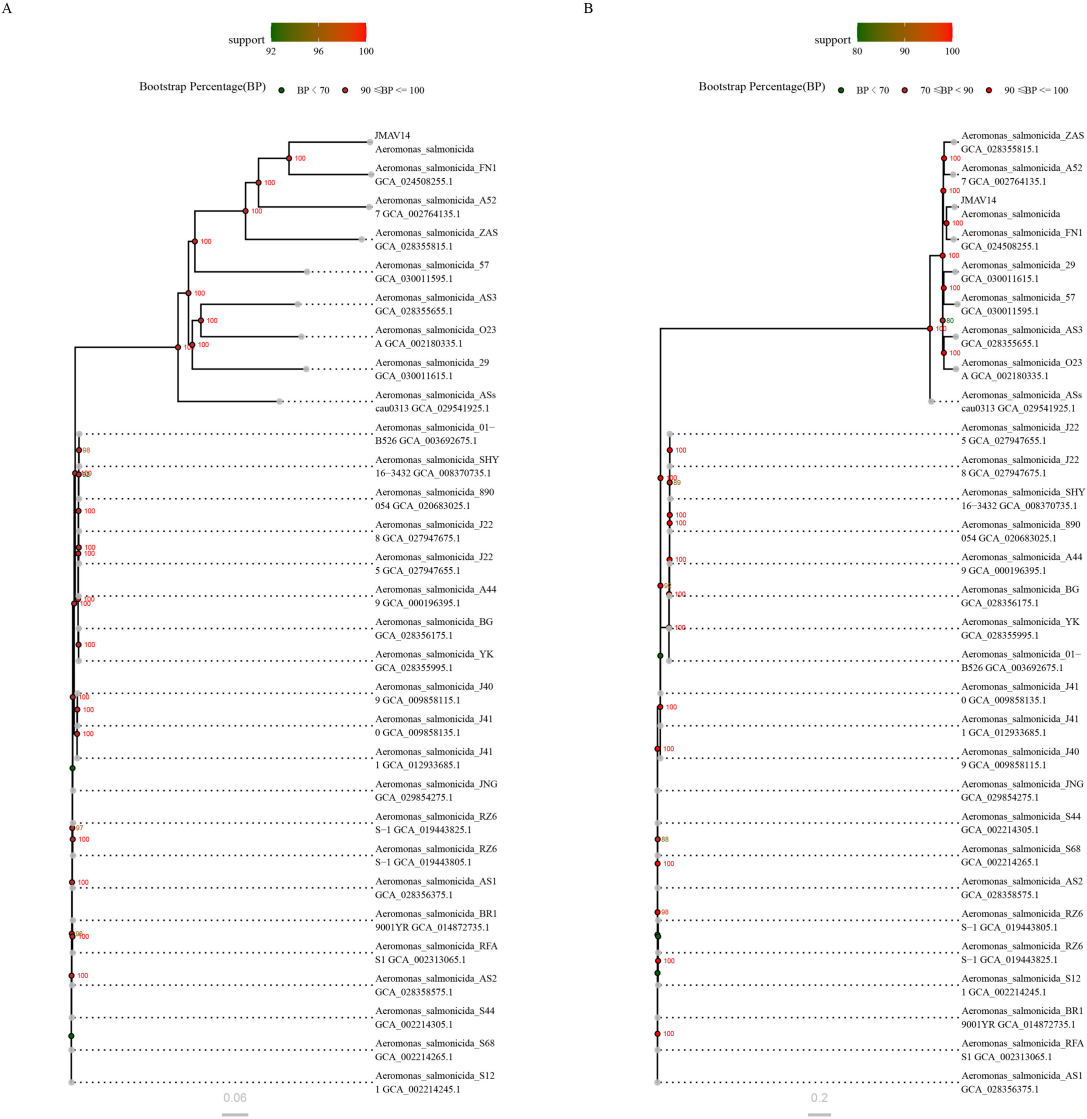
**(A)** Circular phylogenetic tree based on single-copy gene sets; **(B)** circular phylogenetic tree based on SNPs.

A phylogenetic tree was constructed using single-copy core gene sets with the Neighbor-Joining (NJ) clustering method, and 1,000 bootstrap replicates were performed to verify the reliability of the branches. The colors of the dots on the branches represent bootstrap support values (BP): green (BP < 70), orange (70 ≤ BP < 90), and red (90 ≤ BP ≤ 100). The BP value of the clade containing JMAV14 reached 100, indicating extremely high reliability of its clustering relationship with homologous strains. The scale bar of “2e−4” represents the nucleotide substitution rate corresponding to branch lengths, which reflects the genetic divergence of single-copy gene sequences. The clustering results showed that JMAV14 clustered together with strain FN1, suggesting a close phylogenetic relationship between them (Figure 11A).

A phylogenetic tree was built based on whole-genome single-nucleotide polymorphism (SNP) loci, with the same clustering method and bootstrap parameters as those for the core genome tree. Branch support values showed that the BP values of all clustering nodes between JMAV14 and homologous strains were ≥90, further verifying the stability of their phylogenetic relationships. The scale bar of “1e−4” corresponds to the genetic divergence of SNP loci. A comparison with the core genome tree revealed that the clustering topologies generated by the two methods were highly consistent, and JMAV14 was consistently grouped into the same clade as strain FN1. These results demonstrated that the phylogenetic position of JMAV14 is stable and reliable regardless of whether conserved genes or whole-genome SNPs are used for analysis (Figure 11B).

### 3.9 Results of mouse pathogenicity assays

**Figure 12:**
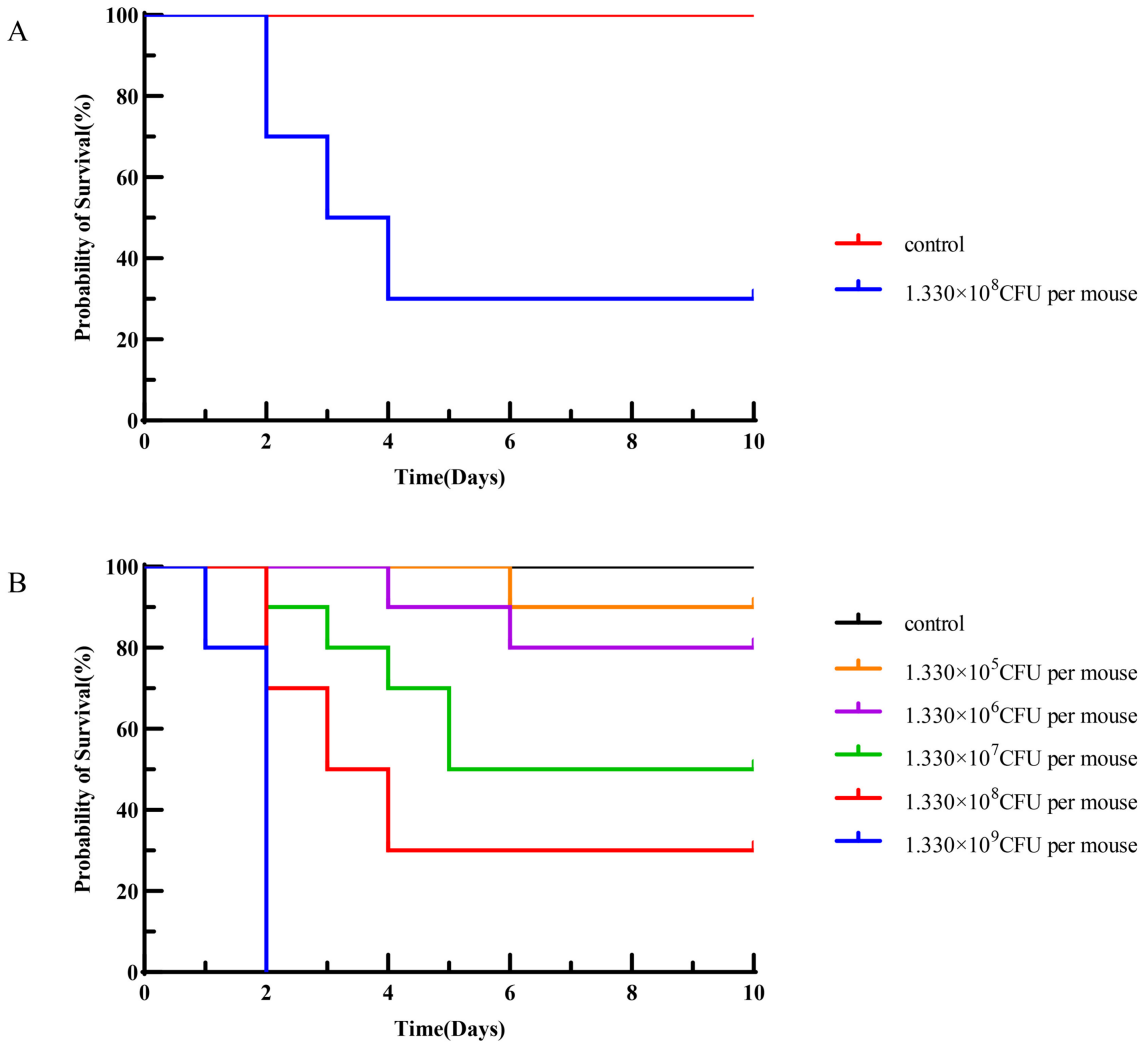
**(A)** Survival curve of mouse pathogenicity assay; **(B)** survival curve of mouse median lethal dose (LD_50_) assay.

As shown in Table 4, the morbidity and mortality rates of mice in the infected group reached 100 and 70%, respectively, while no morbidity was observed in the control groups. Chi-square test demonstrated an extremely significant difference in mortality between the infected group and control groups ( $x^{2}$ = 46.67, *p* < 0.001). The survival curve of the mouse pathogenicity assay (Figure 12A) showed that mice in the infected group began to die on the 2nd day post-infection, with a mortality rate of 70% and a final survival rate stabilized at 30% during the 10-day observation period. In contrast, no deaths occurred in the control groups throughout the experiment, with their survival rate remaining at 100%. Log-rank test confirmed an extremely significant difference in survival between the infected group and control groups ( $x^{2}$ = 28.74, *df* = 1, *p* < 0.001). Combined with the 100% morbidity rate presented in Table 4, these results clearly indicated that JMAV14 is highly pathogenic to mice.

**Table 4:** Results of mouse pathogenicity assay.

Group	Number of mice	Bacterial suspension concentration (CFU/mL)	Inoculation volume (mL)	Actual infection dose (CFU/mouse)	Morbidity rate (%)	Mortality rate (%)	Bacteriological detection positive rate (%)
Infection group	10	3.326 × 10^8^	0.4	1.330 × 10^8^	100	70	100
Negative control group	10	Sterile physiological saline	0.4	—	0	0	0
Blank control group	10	—	—	—	0	0	0

According to the data in Table 5, the median lethal dose (LD₅₀) was calculated to be 1.330 × 10^7^ CFU per mouse, with a 95% confidence interval (95% CI) of 1.248 × 10^7^–1.416 × 10^7^ CFU per mouse. The survival curve of the mouse LD₅₀ assay exhibited a significant dose-dependent pattern (Figure 12B): all mice in the high-dose group (1.330 × 10^9^ CFU per mouse) died within 2 days, with the survival rate dropping rapidly to 0; the mortality rate of the medium-dose group (1.330 × 10^8^ CFU per mouse) was 70%, and the survival rate stabilized at 30% after 5 days; the mortality rate of the low-dose group (1.330 × 10^5^ CFU per mouse) was only 10%, showing no significant difference in survival rate compared with the control groups. Log-rank test revealed extremely significant differences in survival between each dose group and the control groups ( $x^{2}$ = 52.36, *df* = 5, *p* < 0.001). Based on the survival curve data, the LD₅₀ of JMAV14 against mice was calculated to be 1.330 × 10^7^ CFU per mouse (95% CI: 1.25 × 10^7^–1.42 × 10^7^ CFU per mouse) using the Karber’s method.

**Table 5:** Results of mouse median lethal dose (LD₅₀) assay.

Group	Number of mice	Bacterial suspension concentration (CFU/mL)	Inoculation volume (mL)	Actual infection dose (CFU/mouse)	Mortality rate (%)	Bacteriological detection positive rate (%)
Infected group	10	3.326 × 10^9^	0.4	1.330 × 10^9^	100	100
	10	3.326 × 10^8^	0.4	1.330 × 10^8^	70	100
	10	3.326 × 10^7^	0.4	1.330 × 10^7^	50	100
	10	3.326 × 10^6^	0.4	1.330 × 10^6^	20	100
	10	3.326 × 10^5^	0.4	1.330 × 10^5^	10	100
Negative control group	10	Sterile physiological saline	0.4	—	0	0
Blank control group	10	—	—	—	0	0

Bacterial isolation and identification were performed on the liver tissues of dead mice from the infected group. The results showed that all the isolated strains were *Aeromonas salmonicida*, with a 100% positive rate of bacteriological detection.

### 3.10 Results of bacterial load detection in mouse organs

JMAV14 colonization was detected in all target organs of mice in the infected group at different time points, whereas no target bacteria were detected in any organs of the negative control group and blank control group. The detailed results are presented as follows:

#### 3.10.1 Dynamic changes in bacterial load in various organs

24 h post-infection: obvious bacterial colonization was observed in all organs. Among these organs, the liver exhibited the highest bacterial load, reaching (1.21 ± 0.15) × 10^6^ CFU/g, followed by the spleen with (8.53 ± 0.92) × 10^5^ CFU/g. The bacterial loads in the lungs and kidneys were relatively lower, at (5.36 ± 0.61) × 10^5^ CFU/g and (4.82 ± 0.57) × 10^5^ CFU/g, respectively.

48 h post-infection: the bacterial loads in all organs increased significantly and reached their peak values. The bacterial load in the liver increased to (1.85 ± 0.21) × 10^7^ CFU/g, while those in the spleen, lungs, and kidneys were (1.32 ± 0.17) × 10^7^ CFU/g, (9.68 ± 1.05) × 10^6^ CFU/g, and (8.94 ± 0.98) × 10^6^ CFU/g, respectively. All these values showed statistically significant differences compared with those at 24 h post-infection (*p* < 0.05).

72 h post-infection: the bacterial loads in all organs decreased slightly compared with those at 48 h post-infection but remained at a relatively high level. The bacterial load in the liver was (1.52 ± 0.18) × 10^7^ CFU/g, and those in the spleen, lungs, and kidneys were (1.05 ± 0.13) × 10^7^ CFU/g, (7.83 ± 0.86) × 10^6^ CFU/g, and (7.15 ± 0.82) × 10^6^ CFU/g, respectively. These values were still significantly higher than those at 24 h post-infection (*p* < 0.05).

#### 3.10.2 Characteristics of bacterial load differences among organs

There were significant differences in bacterial loads among different organs. The liver and spleen were consistently the main colonization organs, with their bacterial loads at all time points significantly higher than those in the lungs and kidneys (*p* < 0.05). These results indicated that JMAV14 has a stronger tissue tropism for the liver and spleen of mice.

### 3.11 Observation of clinical symptoms and pathological changes in mice

The clinical symptoms of sick mice were as follows: messy fur, depression, sluggish response, huddling together to avoid cold, eyelid adhesion, profuse eye discharge, and reduced food intake.

**Figure 13:**
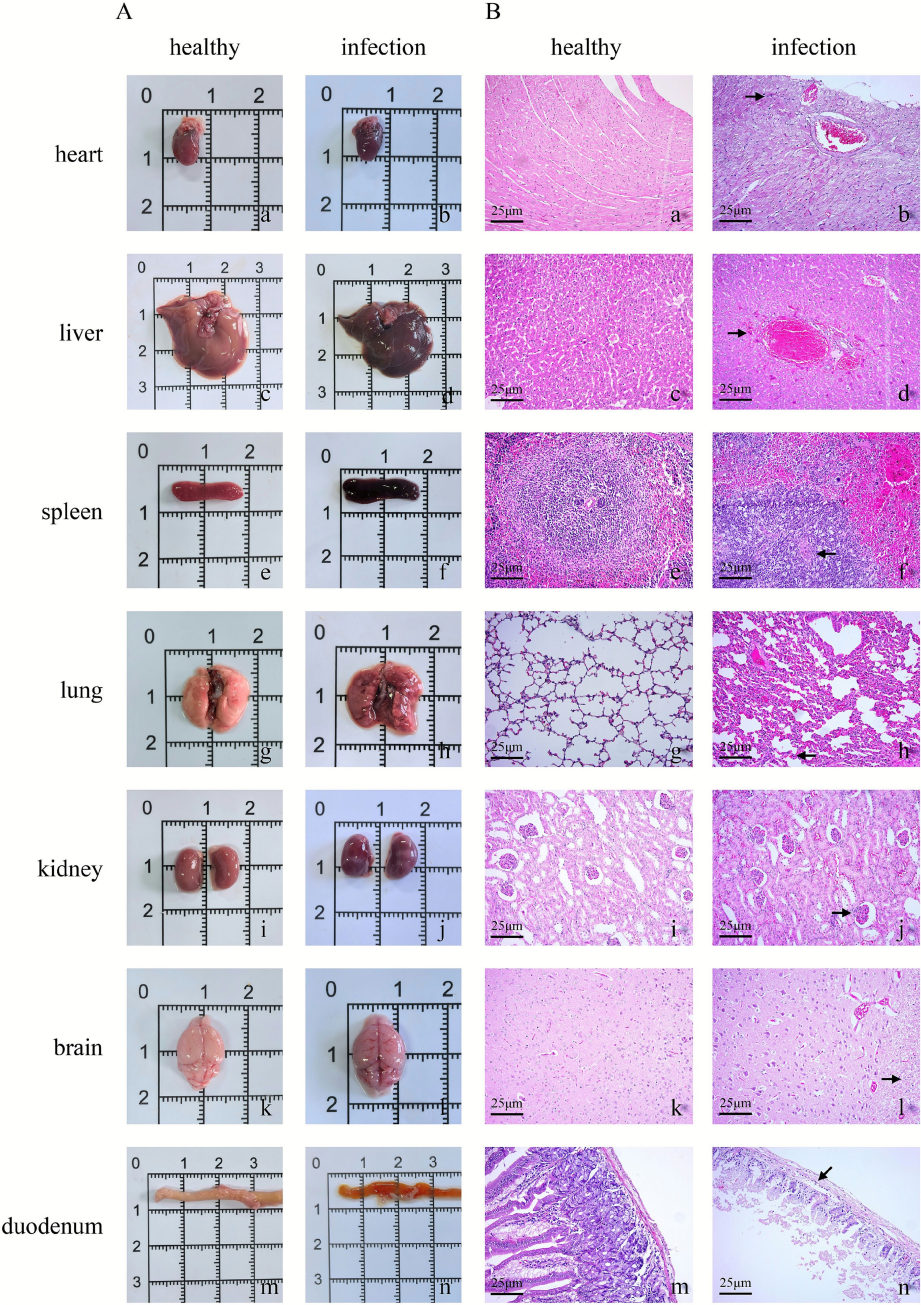
**(A)** Necropsy images of mice; **(B)** hematoxylin–eosin (H&E) stained sections of different mouse organs (200×). The “25 μm” label at the bottom left corner of all sections represents the scale bar.

Necropsy findings of dead mice showed: cardiac congestion and hemorrhage (Figure 13A-b); uneven liver color, congestion and hemorrhage (Figure 13A-d); splenomegaly, congestion and hemorrhage (Figure 13A-f); uneven lung color, fleshy consolidation and hemorrhage (Figure 13A-h); renal swelling, uneven color, congestion and hemorrhage (Figure 13A-j); meningeal congestion (Figure 13A-l); and flushing of the duodenal mucosa (Figure 13A-n). Necropsy images of healthy mice in the control group (Figures 13A-a, 13A-c, 13A-e, 13A-g, 13A-i, 13A-k, 13A-m).

Results of histopathological observation of dead mice demonstrated: Dilation and congestion of myocardial blood vessels, disappearance of myocardial fiber striations, and neutrophil infiltration (Figure 13B-b). Dilation and congestion of hepatic central veins, arteries and interlobular veins, swelling and granular degeneration of hepatocytes, and neutrophil infiltration (Figure 13B-d). Reduction in the number of lymphocytes in splenic corpuscles, necrosis and disintegration of lymphocytes and reticular cells in the medulla, and macrophage infiltration (Figure 13B-f). Interstitial pneumonia, with significant widening of alveolar septa, dilation and congestion of capillaries in the septa, and massive monocyte infiltration (Figure 13B-h). Interstitial nephritis, with swelling of renal tubular epithelial cells, stenosis and occlusion of tubular lumens, and dilation and congestion of small blood vessels (Figure 13B-j). Satellitosis in the brain and massive monocyte infiltration (Figure 13B-l). Necrosis and shedding of duodenal mucosal epithelium, increased number of secretory cells, incomplete glandular epithelium, leukocyte infiltration, and dilation and congestion of small blood vessels (Figure 13B-n). Histopathological observation images of healthy mice in the control group (Figures 13B-a, 13B-c, 13B-e, 13B-g, 13B-i, 13B-k, 13B-m).

### 3.12 Results of fish pathogenicity assay

As shown in Table 6, the morbidity and mortality rates of fish in the infected group reached 100 and 50%, respectively, while no morbidity was observed in the control groups. Chi-square test demonstrated an extremely significant difference in mortality between the infected group and control groups ( $x^{2}$ = 16.67, *p* < 0.001).

**Table 6:** Results of fish pathogenicity assay.

Group	Number of fish	Bacterial suspension concentration (CFU/mL)	Inoculation volume (mL)	Actual infection dose (CFU/fish)	Morbidity rate (%)	Mortality rate (%)	Bacteriological detection positive rate (%)
Infected group	10	3.326 × 10^7^	0.3	9.978 × 10^6^	100	50	100
Negative control group	10	Sterile physiological saline	0.3	—	0	0	0
Blank control group	10	—	—	—	0	0	0

**Figure 14:**
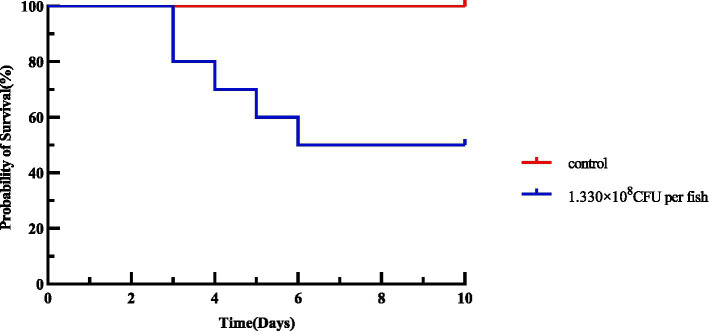
Survival curve of fish pathogenicity assay.

In the fish pathogenicity assay, the survival curve of the infected group (9.978 × 10^6^ CFU/fish) showed a significant difference from that of the control group (Figure 14). Fish in the infected group began to die on the 3rd day post-infection, with a mortality rate of 50% and a survival rate decreased to 50% during the 10-day observation period. No morbidity or mortality was observed in the control group throughout the experiment, with the survival rate remaining at 100%. Log-rank test revealed an extremely significant difference in survival between the two groups ( $x^{2}$ = 16.67, *df* = 1, *p* < 0.001). Combined with the 100% morbidity rate presented in Table 6, these results confirmed that JMAV14 has cross-species pathogenicity.

Bacterial isolation and identification were performed on the liver tissues of dead fish from the infected group. The results showed that all the isolated strains were *Aeromonas salmonicida*, with a 100% positive rate of bacteriological detection.

### 3.13 Observation of clinical symptoms in infected fish

**Figure 15:**
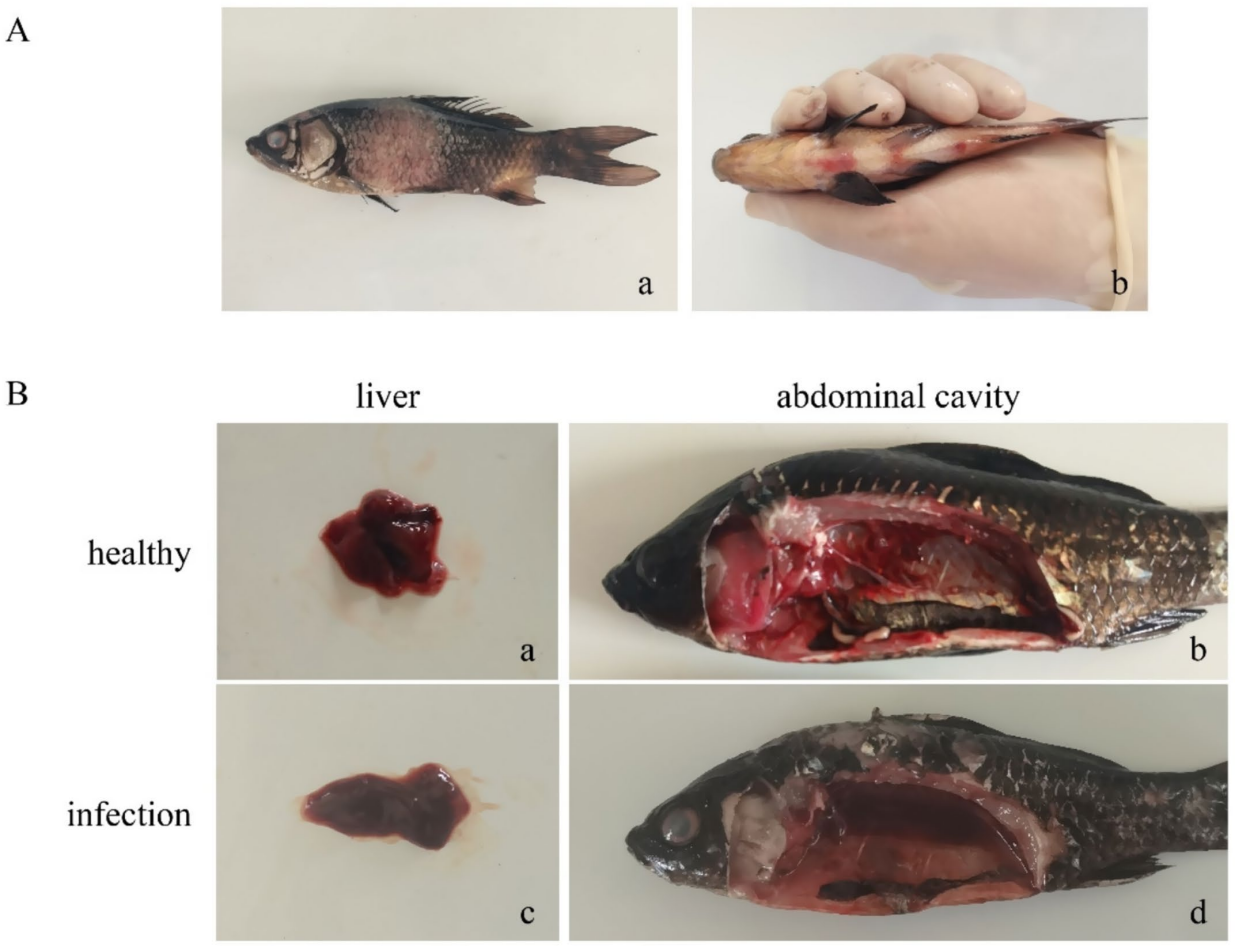
**(A)** Clinical symptom images of infected fish; **(B)** necropsy images of infected fish.

The main clinical symptoms of infected fish were manifested as: scale loss, as well as hemorrhage at the pectoral fins, pelvic fins and anus (Figures 15A-a,b). Necropsy findings of dead fish showed: hepatic congestion, hemorrhage and friable texture (Figures 15B-a,c); a large amount of hemorrhagic ascites in the abdominal cavity (Figures 15B-b,d).

### 3.14 Results of bacterial load detection in fish organs

Stable colonization of JMAV14 was detected in all target organs of infected *Carassius carassius* at different time points, whereas no target bacteria were detected in any organs of the negative control group and blank control group. The detailed results are presented as follows:

#### 3.14.1 Dynamic changes in bacterial load in various organs

24 h post-infection: obvious bacterial colonization was observed in all target organs. Among these organs, the liver exhibited the highest bacterial load, reaching (6.23 ± 0.71) × 10^5^ CFU/g, followed by the kidney with (4.81 ± 0.53) × 10^5^ CFU/g. The bacterial load in the gill tissue was (3.15 ± 0.38) × 10^5^ CFU/g.

48 h post-infection: the bacterial loads in all organs increased significantly and reached their peak values. The bacterial load in the liver increased to (1.56 ± 0.19) × 10^6^ CFU/g, while those in the kidney and gill tissue were (1.12 ± 0.14) × 10^6^ CFU/g and (7.83 ± 0.85) × 10^5^ CFU/g, respectively. All these values showed statistically significant differences compared with those at 24 h post-infection (*p* < 0.05).

72 h post-infection: The bacterial loads in all organs decreased slightly compared with those at 48 h post-infection but remained at a relatively high level. The bacterial load in the liver was (1.28 ± 0.16) × 10^6^ CFU/g, and those in the kidney and gill tissue were (9.57 ± 1.02) × 10^5^ CFU/g and (6.42 ± 0.73) × 10^5^ CFU/g, respectively. These values were still significantly higher than those at 24 h post-infection (*p* < 0.05).

#### 3.14.2 Characteristics of bacterial load differences among organs

There were significant differences in bacterial loads among different organs. The liver and kidney were consistently the main colonization organs, with their bacterial loads at all time points significantly higher than those in the gill tissue (*p* < 0.05). These results indicated that JMAV14 has a stronger tropism for the liver and kidney tissues of *Carassius carassius*, which is consistent with the common pathogenic characteristics of bacterial diseases in fish.

### 3.15 Results of antimicrobial susceptibility testing

The results of antimicrobial susceptibility testing showed that JMAV14 was susceptible to ceftazidime, meropenem, gentamicin, trimethoprim-sulfamethoxazole and colistin, but resistant to tetracycline (Table 7).

**Table 7:** Antimicrobial susceptibility testing results of strain JMAV14.

Drug class	Drug name	Concentration (mg/L)	Interpretation threshold (mg/L)			Reference standard	Result
			R	I	S		
β-lactams	Ampicillin	64	≥32.00	16.00	≤8.00	CLSI M100-Ed34	—
	Amoxicillin	8	≥32.00	16.00	≤8.00	CLSI M100-Ed34	—
	Clavulanate Potassium	4	≥32.00	16.00	≤8.00	CLSI M100-Ed34	—
	Ceftiofur	2	≥8.00	4.00	≤2.00	CLSI M100-Ed34	—
	Ceftazidime	<0.12	≥16.00	8.00	≤4.00	CLSI M45-A3	S
	Meropenem	0.03	≥4.00	2.00	≤1.00	CLSI M45-A3	S
Aminoglycosides	Gentamicin	1	≥16.00	8.00	≤4.00	CLSI M45-A3	S
	Spectinomycin	>512	≥128.00	64.00	≤32.00	CLSI M100-Ed34	—
	Apramycin	4	≥16.00	8.00	≤4.00	CLSI M100-Ed34	—
Tetracyclines	Tetracycline	16	≥16.00	8.00	≤4.00	CLSI M45-A3	R
Phenicols	Florfenicol	2	≥16.00	8.00	≤4.00	CLSI M100-Ed34	—
Sulfonamides	Sulfisoxazole	512	≥512.00	—	≤256.00	CLSI M100-Ed34	—
	Trimethoprim-Sulfamethoxazole	0.12–2.4	≥4.00–76.00	—	≤2.00–38.00	CLSI M45-A3	S
Quinolones	Enrofloxacin	0.12	≥2.00	0.50–1.00	≤0.25	CLSI M100-Ed34	—
	Ofloxacin	0.25	≥8.00	4.00	≤2.00	CLSI M100-Ed34	—
Quinoxalines	Mequindox	4	—	—	≤4.00	CLSI M100-Ed34	—
Polypeptides	Colistin	<0.12	≥4.00	—	≤2.00	CLSI M100-Ed34	S

CLSI M45-A3 is the exclusive interpretive standard for the genus *Aeromonas*. In this study, only this standard was used to determine the susceptibility (S)/resistance (R) of the corresponding antimicrobial agents. CLSI M100-Ed34 is a general interpretive standard for aerobic Gram-negative bacteria, which only provides reference thresholds without susceptibility/resistance determination (marked as “—”). R denotes resistance, I denotes intermediate susceptibility, S denotes susceptibility, and “—” indicates no determination result or no corresponding threshold.

## 4 Discussion

*Aeromonas salmonicida* is a psychrophilic, non-motile, and facultatively anaerobic Gram-negative bacterium that is widely distributed in marine and freshwater environments (Wang et al., 2022). First isolated from salmon by Emmerich and Weibel in 1894 (Hussain Bhat et al., 2021), *Aeromonas salmonicida* has been classified into five subspecies to date, including four psychrophilic subspecies (*Aeromonas salmonicida* subsp. *salmonicida*, *Aeromonas salmonicida* subsp. *achromogenes*, *Aeromonas salmonicida* subsp. *masoucida*, *Aeromonas salmonicida* subsp. *smithia*) and one mesophilic subspecies (*Aeromonas salmonicida* subsp. *pectinolytica*) (Keeling et al., 2013). Slight differences exist in the morphological and biochemical characteristics among different subspecies. Based on traits such as pigment production ability, colony size, growth rate, hemolytic activity, and sucrose fermentation capacity, *Aeromonas salmonicida* subsp. *salmonicida* is generally designated as the typical strain, while other subspecies and strains that do not conform to the subspecies characteristics are referred to as atypical strains (Reith et al., 2008). The JMAV14 strain isolated in this study did not exhibit all the characteristics of the aforementioned typical strain, and thus it should be classified as an atypical strain. Results of the biochemical identification in this study showed a high similarity between JMAV14 and *Aeromonas salmonicida* subsp. *pectinolytica* as described in *Bergey’s Manual of Determinative Bacteriology* and the study by Pavan ME *et al*. Therefore, JMAV14 is speculated to be *Aeromonas salmonicida* subsp. *pectinolytica*.

The 16S rRNA gene sequence is highly conserved during phylogeny and has been widely used for bacterial species and genus identification. The 16S rRNA phylogenetic tree constructed in this study showed that JMAV14 clustered into the same clade with known strains of *Aeromonas salmonicida*. The A-layer protein encoded by the *VapA* gene is an important virulence protein of *Aeromonas salmonicida* (Lago et al., 2012), and the *VapA* gene serves as a crucial criterion for the typing and subspecies identification of *Aeromonas salmonicida* (Gulla et al., 2016). Based on the variable regions of the *VapA* gene, *Aeromonas salmonicida* is classified into 14 subtypes, in addition to *Aeromonas salmonicida* subsp. *pectinolytica* which lacks the *VapA* gene (Gulla et al., 2019). In this study, PCR amplification of the *VapA* gene yielded no target band, indicating that JMAV14 does not harbor the *VapA* gene, a result that is most consistent with the characteristics of *Aeromonas salmonicida* subsp. *pectinolytica*. An average nucleotide identity (ANI) value of 95–96% between the genomes of two strains is equivalent to a DNA–DNA hybridization (DDH) value of 70% and a 16S rRNA gene similarity of 98.65%. Therefore, two strains with a genomic ANI value >96% are identified as the same species, while those with an ANI value <95% are recognized as different species (Michael and Ramon, 2009). The ANI analysis results in this study revealed that JMAV14 shared the highest ANI value (98.08%) with strain FN1, which is annotated as *Aeromonas salmonicida* in the NCBI database. These findings confirmed that JMAV14 belongs to *Aeromonas salmonicida* at the species level from a genomic perspective, and simultaneously indicated that JMAV14 has the closest phylogenetic relationship with strain FN1. In summary, this study confirmed the species classification of JMAV14 using the 16S rRNA phylogenetic tree (at the single conserved gene level) and ANI analysis (at the whole-genome level). Its subspecies classification was further verified by *VapA* gene detection and biochemical characteristics. Collectively, these results demonstrated that JMAV14 is *Aeromonas salmonicida* subsp. *pectinolytica*.

Virulence factor prediction indicated that the virulence factors of strain JMAV14 cover the complete pathogenic pathway of *motility-colonization-toxin release-nutrient acquisition*. Among these factors, the high proportion of proteins associated with motility, chemotaxis, and type IV pilus assembly constitutes the key molecular basis for its strong pathogenicity and cross-species infectivity.

Comprehensive analysis of antimicrobial resistance (AMR) gene prediction and antimicrobial susceptibility testing results revealed the molecular characteristics and potential mechanisms underlying the drug-resistant phenotype of strain JMAV14: the high minimum inhibitory concentration (MIC) value of sulfisoxazole (512 mg/L) was highly consistent with the presence of the *sul1* gene (100% homology). This gene mediates sulfonamide resistance through target substitution, acting as the core driver gene. The tetracycline-resistant phenotype (categorized as resistant [R] per CLSI M45-A3 criteria) is speculated to be mediated by the cross-resistance of the *qacEdelta1* efflux pump. Although no typical *tet* genes were identified, the broad-spectrum substrate specificity of the major facilitator superfamily (MFS) efflux pump can explain this resistant phenotype. The susceptible phenotypes to meropenem and gentamicin were inconsistent with the presence of the *imiS* and *AAC(6′)-Ib4* resistance genes, which might be attributed to the loss of enzyme activity caused by key site mutations in these genes, or the offsetting effect of enhanced drug uptake on resistance. Susceptibility to florfenicol was associated with the low substrate affinity of the catB3 enzyme. The high MIC values of spectinomycin (MIC > 512 mg/L) and ampicillin (MIC = 64 mg/L) suggest potential drug resistance; however, no directly corresponding resistance genes were identified. This may be due to species-specific genetic variations or gene omission caused by overly stringent screening thresholds, which requires validation via combined annotation using multiple databases. In clinical practice, the use of sulfonamides and tetracyclines should be avoided, and susceptible drugs such as meropenem and gentamicin should be prioritized. Genes such as *sul1* are often located on mobile genetic elements, so vigilance is required against their dissemination via horizontal gene transfer, which may exacerbate the risk of resistance spread in aquaculture environments. In this study, the susceptibility results of drugs without dedicated breakpoints are for *in vitro* reference only, and practical clinical medication should be comprehensively determined based on the scenario of strain infection and the metabolic characteristics of the drugs.

To date, *Aeromonas salmonicida* has been mostly reported to infect aquatic organisms, humans, and some mammals, with no documented cases of bovine infection. Furthermore, among these mammalian infection cases, the pathogen has not been clearly identified as *Aeromonas salmonicida* subsp. *pectinolytica*. Pavan et al. (2000) first isolated *Aeromonas salmonicida* subsp. *pectinolytica* from a heavily polluted river in Argentina in 2000. Upon literature retrieval, there are currently no reports on the isolation of pathogenic *Aeromonas salmonicida* subsp. *pectinolytica* strains from the tissues or organs of diseased animals.

This study confirmed that strain JMAV14 is pathogenic to experimental mice (70% mortality at a dose of 1.330 × 10^8^ CFU/mouse) and *Carassius carassius* (50% mortality at a dose of 9.978 × 10^6^ CFU/fish), with a 100% positive rate of bacteriological detection in both hosts, indicating its cross-species pathogenic potential to these two experimental hosts. In addition, this study only verified pathogenicity under laboratory conditions; the transmission capacity and actual risks of JMAV14 in natural aquaculture environments still need to be further confirmed by combining field survey data.

In prokaryotes, the CRISPR system mediates an adaptive immune response by integrating DNA fragments homologous to those of phages, viruses, or exogenous plasmids into its spacer sequences, thereby acquiring the ability to specifically eliminate the DNA of invading phages, viruses, or plasmids. Meanwhile, the integration of DNA fragments from diverse sources into spacer sequences enables bacteria to achieve diversity in CRISPR-mediated adaptive immunity. Furthermore, since new spacer sequences are directly integrated into the bacterial genome, this immune trait is preserved during cell division and ultimately confers immune memory (Zheng et al., 2016). The integration of temperate phage nucleic acids into the host genome gives rise to prophages, and bacteria harboring prophages in their genomes are defined as lysogenic bacteria. The presence of prophage sequences may enable certain bacteria to acquire antibiotic resistance, enhance their environmental adaptability, improve their adhesion capacity, or even convert them into pathogenic strains (Wang, 2023). In this study, we characterized the prophage profiles of strain JMAV14 and confirmed the absence of a CRISPR system. However, no direct evidence of an association between prophages and virulence genes was identified, precluding the verification of their roles in virulence evolution. Due to the lack of experimental data, such as prophage gene knockout assays and virulence phenotype validation, the functional significance of these prophages cannot be inferred, which represents one of the limitations of this study. Future research could validate prophage functions through *in vitro* experiments to clarify their relationship with the pathogenicity of the strain. In addition, JMAV14 carries five prophages, leading to the inference that these prophages may constitute a key factor underlying the infection of cattle by *Aeromonas salmonicida*. Moreover, the horizontal transfer of virulence genes is not mediated by a single mechanism; besides prophages, plasmids or transposons may also be involved in the acquisition and dissemination of virulence genes. Future studies will further isolate and identify the plasmid components of JMAV14, and combine these findings with horizontal gene transfer analyses to elucidate the origin and transmission pathways of its pathogenicity-related genes.

In this study, phylogenetic trees were constructed using two complementary approaches, namely the single-copy core gene set and whole-genome SNP analysis. The topological structures of the two trees showed a high degree of consistency, which reflects the stability of the intraspecific genetic relationships within *Aeromonas salmonicida*. Based on the clustering results, JMAV14 and strain FN1 stably clustered into the same clade with a bootstrap support value of 100. This suggests that JMAV14 and FN1 diverged relatively recently during evolution and share a close phylogenetic relationship. In summary, the phylogenies validated by the dual-method approach clearly delineate the phylogenetic position of JMAV14 within *Aeromonas salmonicida*. The close genetic affinity between JMAV14 and FN1 provides a solid genetic background for subsequent functional studies.

The median lethal dose (LD₅₀) of JMAV14 in mice was determined to be 1.330 × 10^7^ CFU per mouse, with a morbidity rate of 100% and a mortality rate of 70%. This indicates stronger pathogenicity than that of the porcine-derived *Aeromonas salmonicida* strain reported by Deng et al. (2019). Histopathological examination of lung tissues from deceased mice revealed significant widening of alveolar septa, dilation and congestion of capillaries within the septa, as well as extensive infiltration of monocytes and segmented neutrophils. These pathological changes showed high similarity to those induced by the caprine-derived *Aeromonas salmonicida* strain described by Wang et al. (2018). This study verified the cross-species pathogenicity of JMAV14 using dual models (mice and fish), demonstrating notable innovation and scientific value: In the mouse model: Most previously reported *Aeromonas salmonicida* subsp. *pectinolytica* strains isolated from environmental samples are non-pathogenic. This study is the first to confirm that this subspecies can cause fatal systemic infections in mammals (mice). Its LD₅₀ value (1.330 × 10^7^ CFU per mouse) reflects significantly higher virulence than that of some porcine- and caprine-derived *Aeromonas salmonicida* strains. Additionally, this is the first confirmation that *Aeromonas salmonicida* subsp. *pectinolytica* can infect mice and *Carassius carassius*, providing new insights into the host range of this subspecies. Detection of bacterial loads in mouse organs clarified the colonization and proliferation characteristics of JMAV14 *in vivo*. Detectable bacterial loads were observed in all target organs at 24 h post-infection, reached peak levels at 48 h, and then maintained a high-load state. This dynamic change was highly consistent with the results of the mouse pathogenicity assay, where obvious clinical symptoms emerged at 24–48 h post-infection and the mortality peak occurred at 48–72 h. These findings confirm that the efficient in vivo proliferation of the strain is the key mechanism underlying mouse morbidity and mortality. The liver and spleen were identified as the primary colonization organs, with bacterial loads significantly higher than those in other organs. This is consistent with the pathogenic characteristics of *Aeromonas salmonicida*—as an intracellular parasitic bacterium, it tends to colonize and proliferate in immune-related organs such as the liver and spleen, leading to host death by impairing immune cell function and triggering systemic inflammatory responses. Sustained invasion of target organs by high bacterial loads causes tissue damage and physiological dysfunction, which further corroborates the strong pathogenicity of JMAV14 and forms complementary validation with the LD₅₀ results (1.330 × 10^7^ CFU per mouse). In the fish model: *Carassius carassius*, a traditional host of *Aeromonas salmonicida*, was selected for cross-species infection verification. The results confirmed that JMAV14 can not only infect terrestrial mammals but also break through host barriers to infect aquatic animals. A mortality rate of 50% and typical septicemic lesions (e.g., fin hemorrhage, massive hemorrhagic ascites in the abdominal cavity) further verified its broad-spectrum pathogenicity. The complementary validation using dual models provides a more comprehensive experimental basis for elucidating the cross-species infection mechanism of this bovine-derived strain. *Aeromonas salmonicida* commonly infects fish and is typically classified into acute, subacute, and chronic forms: Subacute or chronic infections mostly occur as furunculosis in aged fish, with typical symptoms including mild exophthalmos, hemorrhage in fins and muscles, and furuncles on the skin or muscle tissues; visceral lesions include hemorrhage in the hepatopancreas, splenomegaly, and renal necrosis. Acute infections often progress rapidly with inconspicuous external symptoms and high mortality rates, predominantly affecting juvenile and adult fish. Typical manifestations include septicemia, melanosis, hemorrhage at the base of fins, and hemorrhage in visceral organs such as the abdominal wall and heart (Wang, 2023). In this study, the fish pathogenicity assay results indicated an acute infection pattern. Infected fish exhibited multiple lesions including fin hemorrhage, massive hemorrhagic ascites in the abdominal cavity, and hepatic hemorrhage, with a morbidity rate of 100% and a mortality rate of 50%. The dynamic changes in bacterial loads in fish organs showed a precise correlation with the results of the pathogenicity assay: bacterial colonization was detected in all organs at 24 h post-infection, and the bacterial loads peaked at 48 h post-infection. This pattern corresponded well with the observation in the fish pathogenicity assay that “distinct clinical symptoms emerged at 48 h post-infection and the mortality rate stabilized after 72 h post-infection,” confirming that the efficient *in vivo* proliferation of strain JMAV14 is the core mechanism underlying the morbidity and mortality of infected fish. As the core organ responsible for metabolism and immunity in fish, the liver exhibited the highest bacterial load, indicating that the strain preferentially invades metabolically active tissues. The strain induces host death by impairing the liver’s detoxification function and triggering systemic inflammatory responses. The kidney, another vital immune organ in fish, sustained high-level bacterial colonization, which inhibits the activity of immune cells, reduces the anti-infection capacity of the fish, and further exacerbates disease progression.

JMAV14 induced acute infections in mice and fish, characterized by high morbidity and mortality rates as well as multiple organ pathological damage, which proves that the bovine-derived *Aeromonas salmonicida* possesses strong pathogenicity. Therefore, high priority should be attached to the prevention and control of *Aeromonas salmonicida* in the animal breeding industry and animal-derived food industry chain, and measures should be taken to prevent its transmission to humans. This study provides guidance for the identification, diagnosis, and development of prevention and control strategies for *Aeromonas salmonicida* subsp. *pectinolytica*, and it also holds great significance for enriching the biological characteristics and theoretical research of *Aeromonas salmonicida* following cross-species infection in different animals.

### 4.1 Study limitations and future perspectives

This study is the first to isolate and identify a pathogenic *Aeromonas salmonicida* subsp. *pectinolytica* strain (JMAV14) from a diseased dead bovine. Combined with whole-genome analysis and dual-host pathogenicity assays, we revealed its cross-species infection characteristics and genetic basis. However, several limitations remain: JMAV14 was isolated from a single diseased bovine sample collected from a cattle farm in Chaoyang, Liaoning Province, as related infection cases in this region are extremely rare. This limitation makes it difficult to generalize the results to reflect the overall prevalence and genetic diversity of this bacterium in cattle populations. The distribution characteristics of this pathogen across cattle farms in different regions and among different cattle breeds remain to be elucidated. Restricted by ethical constraints and experimental conditions, no bovine challenge trials were performed. Thus, direct verification of the clinical pathogenicity and transmission routes of this strain in its original host (cattle) was not achieved. To address these shortcomings, future research will focus on the following aspects: expand the scope of sample collection to multiple cattle farms in Liaoning Province and surrounding areas, systematically isolate strains, and analyze their serotypes, genotypes, and virulence gene polymorphisms to clarify the epidemiological characteristics and genetic diversity of the pathogen. Conduct bovine challenge trials, and combine the analysis of clinical symptoms, pathological changes, and virulence factor expression to verify the pathogenicity of the strain, as well as to elucidate its infection mechanisms and transmission routes. Further investigate the role of mobile genetic elements such as plasmids and transposons in the dissemination of virulence genes, thereby providing a more comprehensive theoretical basis for formulating targeted prevention and control strategies.

## 5 Conclusion

In conclusion, this study employed a polyphasic identification approach to isolate and characterize a bovine-derived atypical strain of *Aeromonas salmonicida* subsp. *pectinolytica* (designated JMAV14) from the liver of a diseased cattle in Chaoyang, Liaoning Province. According to available published research, there were no previous records of pathogenic strains of this subspecies being isolated from the tissues or organs of diseased animals, and this study thus fills the relevant gap in this field. Through genomic analysis and pathogenicity assays, this study clarified the phylogenetic position of *Aeromonas salmonicida* strain JMAV14 (it shares the closest genetic relationship with strain FN1), confirmed its cross-species pathogenicity to mice and *Carassius carassius* under laboratory conditions, and determined its median lethal dose (LD₅₀ = 1.330 × 10^7^ CFU/mouse) in mice. The findings of this study provide basic data for the pathogenic characteristics of strain JMAV14, and also offer a reference for the development of laboratory detection and prevention/control technologies for similar strains.

## Data Availability

The datasets presented in this study can be found in online repositories. The names of the repository/repositories and accession number(s) can be found in the article/Supplementary material.
